# Association between human papillomavirus infection and common sexually transmitted infections, and the clinical significance of different *Mycoplasma* subtypes

**DOI:** 10.3389/fcimb.2023.1145215

**Published:** 2023-03-16

**Authors:** Disi A, Hui Bi, Dai Zhang, Bingbing Xiao

**Affiliations:** Department of Obstetrics and Gynecology, Peking University First Hospital, Beijing, China

**Keywords:** sexually transmitted infection, vaginal microecological disorder, human papillomavirus, cervical intraepithelial neoplasia, *Ureaplasma parvum*, herpes simplex virus type 2

## Abstract

**Introduction:**

Human papillomavirus (HPV) infection, especially persistent high-risk HPV, is associated with cervical cancer. Female reproductive tract microecological disorders and lower genital tract infections have been increasingly correlated with HPV infection and cervical lesions. Due to their common risk factors and transmission routes, coinfection with other sexually transmitted infections (STIs) has become a concern. Additionally, the clinical significance of *Mycoplasma* subtypes appear to vary. This study aimed to assess the correlations between common STIs and HPV infection, and to investigate the clinical significance of *Mycoplasma* subtypes.

**Methods:**

We recruited 1,175 patients undergoing cervical cancer screening at the Peking University First Hospital gynecological clinic from March 2021 to February 2022 for vaginitis and cervicitis tests. They all received HPV genotyping and detection of STIs, and 749 of them underwent colposcopy and cervical biopsy.

**Results:**

Aerobic vaginitis/desquamative inflammatory vaginitis and STIs (mainly single STIs) were found significantly more often in the HPV-positive group than in the HPV-negative group. Among patients with a single STI, rates of infection with herpes simplex virus type 2 or UP6 in the HPV-positive group were significantly higher than in the HPV-negative group (OR_adj_: 1.810, 95%CI: 1.211–2.705, P=0.004; OR_adj_: 11.032, 95%CI: 1.465–83.056, P=0.020, respectively).

**Discussion:**

Through detailed *Mycoplasma* typing, a correlation was found between different *Mycoplasma* subtypes and HPV infection. These findings suggest that greater attention should be paid to detecting vaginal microecological disorders in those who are HPV-positive. Further, lower genital tract infections, including both vaginal infections and cervical STIs, are significantly more common among women who are HPV-positive and who thus require more thorough testing. Detailed typing and targeted treatment of *Mycoplasma* should become more routine in clinical practice.

## Introduction

Human papillomavirus (HPV) is among the most common sexually transmitted infections (STIs), and persistent high-risk HPV (HR-HPV) infection is associated with cervical cancer (Tommasino, 2014). More than 200 HPV subtypes have been found to infect humans, among which 14 (HPV16, 18, 31, 33, 35, 39, 45, 51, 52, 56, 58, 59, 66, and 68) have been identified as carcinogenic (Kombe Kombe et al., 2020; World Health Organization, 2021). More and more studies have shown that genital tract microecological disorder may be associated with HPV infection and cervical lesions (Zeng et al., 2022; Xu et al., 2023).

Genital tract infections, including vaginitis and cervicitis, can lead to genital tract microecological disorders. Common types of vaginitis, including bacterial vaginosis (BV), aerobic vaginitis (AV), desquamative inflammatory vaginitis (DIV), trichomoniasis (TV), vulvovaginal candidiasis (VVC), and cervicitis, are associated with STIs. STIs are a significant medical problem affecting women’s health, with an estimated global daily incidence of 1 million (Unemo et al., 2017). Common causes of STIs include *Chlamydia trachomatis* (CT), *Neisseria gonorrhoeae* (NG), herpes simplex virus type 2 (HSV-2), *Mycoplasma hominis* (MH), *Mycoplasma genitalium* (MG), *Ureaplasma urealyticum* (UU), and *Ureaplasma parvum* (UP). The World Health Organization estimates that CT infection, NG infection, syphilis, and TV together accounted for 357.4 million new infections globally in 2012 (Newman et al., 2015). The increasing prevalence of *Mycoplasma* infections and other STIs, including HSV-2 infection, and rates of antibiotic resistance are also concerning. Although earlier studies merely detected and analyzed “genital *Mycoplasma* infection” (Friedek et al., 2004; Zhang et al., 2010), it is now acknowledged that *Mycoplasma* can be further divided into subtypes with varying clinical significance, including MH, MG, UU, and UP. Among these, MH infection is found to associate with an increased risk of cervicitis, pelvic inflammatory disease, and infertility, whereas MG, UU, and UP can be positive in not only symptomatic but also frequently in healthy individuals (Zhang and Liu, 2016; Horner et al., 2018; Tuddenham et al., 2022).

In this study we recruited subjects undergoing cervical cancer screening and tested them for genital tract infection, including carrying out detailed typing of *Mycoplasma*, to investigate the correlation between common STIs and HPV infection. The aim of the study was to guide more efficient and comprehensive clinical examination and diagnosis.

## Materials and methods

### Study cohort and clinical sample collections

**Figure 1:**
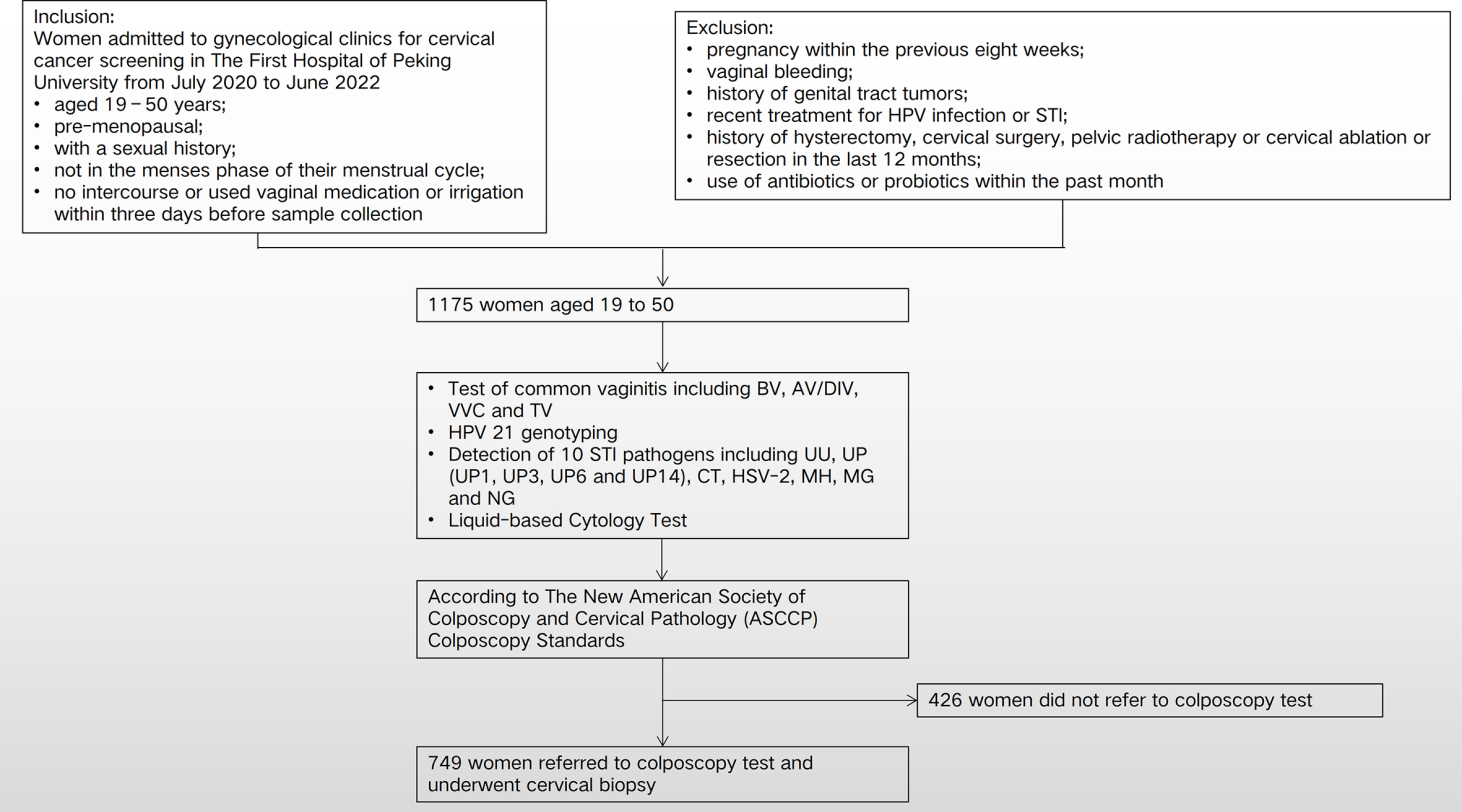
Flow chart and study design. HPV, human papillomavirus; STI, sexually transmitted infection; CT, *Chlamydia trachomatis*; UU, *Ureaplasma urealyticum*; MH, *Mycoplasma hominis*; MG, *Mycoplasma genitalium*; UP, *Ureaplasma parvum*; NG, *Neisseria gonorrhoeae*; HSV-2, herpes simplex virus type 2; BV, bacterial vaginosis; AV/DIV, aerobic vaginitis/desquamative inflammatory vaginitis; TV, trichomoniasis vaginalis; VVC, vulvovaginal candidiasis.

This human study was reviewed and approved by the Ethics Committee of Peking University First Hospital (2021KY062). We collected data on 1,175 subjects who underwent cervical cancer screening at gynecological clinics of The First Hospital of Peking University from March 2021 to February 2022 and who met the following requirements: age 19–50 years; pre-menopausal; sexual history; not in the menses phase of their menstrual cycle; and abstention from intercourse or vaginal medication or irrigation for 3 days before sample collection. Cervical samples were collected from all enrolled subjects for molecular detection of HPV and STI pathogens. Those referred for colposcopy underwent comprehensive colposcopy and pathological biopsy of any abnormal cervical tissue. Exclusion criteria were as follows: pregnancy within the previous 8 weeks; vaginal bleeding; history of genital tract tumors; recent treatment for HPV infection or other STI; a history of hysterectomy, cervical surgery, pelvic radiotherapy, or cervical ablation or resection in the last 12 months; and use of antibiotics or probiotics within the past month (**Figure 1**).

Samples were collected by professional gynecologists after standardized training. With the patient in the lithotomy position, vaginal secretions were collected with a swab at a standard anatomical site (one-third of the way up the lateral vaginal wall) and rolled onto a glass slide for immediate Gram staining to detect candidal hyphae and spores and clue cells. Exposing the cervix with a sterile speculum without lubricant, a sterile cotton swab was rotated five times in the cervical canal at a depth of 1–2 cm for 15–20 seconds before removal. Exfoliated cervical cells and secretions were obtained from the cervical epithelium with two cell brushes and stored at 4°C. They were then transferred to a buffer for DNA testing immediately or within 10 days, for separate genotyping of 21 HPV subtypes and STI pathogen detection.

BV was diagnosed by using the Gram stain-based Nugent score (0–3 was considered BV negative, 4–6 intermediate, and 7–10 BV positive) (Nugent et al., 1991) and, using the modified Amsel diagnostic criteria (Amsel et al., 1983) when three of the following were present: thin homogeneous discharge; a vaginal pH > 4.5; release of amines on addition of 10% potassium hydroxide to vaginal fluid; and the presence of clue cells. VVC was diagnosed by identification of budding yeasts, hyphae, or pseudo-hyphae in a wet preparation (saline, 10% potassium hydroxide) of vaginal discharge, or if Gram staining yielded a positive result for a yeast species. TV was diagnosed by wet mount microscopy immediately following vaginal secretion swab. AV/DIV was diagnosed using the criteria proposed by Donders et al. (Donders et al., 2017), based on lactobacillary grade and the presence of other bacteria, leukocytes, and parabasal epithelial cells.

### HPV genotyping and detection of sexually transmitted pathogens

The 21 HPV GenoArray Diagnostic Kit (HBGA-21PKG; HybriBio, Ltd., Chaozhou, China) was used, with the Rapid Capture System, using a HPV genotyping macroarray for HPV identification. The kit detects 14 HR-HPV types (HPV16, 18, 31, 33, 35, 39, 45, 51, 52, 56, 58, 59, 68, and 66), one suspected HR-HPV type (HPV53), and six low-risk HPV (LR-HPV) types (HPV6, 11, 42, 43, 44, and CP8304). We used the kit in accordance with the manufacturer’s instructions and analyzed the results of cell lysis, DNA extraction, polymerase chain reaction (PCR) amplification, and hybridization.

A nucleic acid detection kit (HBRT-STD6; HybriBio, Ltd.) was used to detect STI pathogens including UU, UP (UP1, 3, 6, and 14), CT, HSV-2, MH, MG, and NG. A real-time PCR fluorescence probe was used to detect the pathogenic STI microorganisms. This method was used for single and mixed infections.

### Liquid-based cytology test

Exfoliated cervical cell specimens were collected by one of two gynecologists using a conical cytobrush, placed into a tube containing 4 mL of preservation solution and temporarily stored at 4°C until testing. Cervical cytology samples were diagnosed using a ThinPrep^®^ cytologic test (TCT) (TriPath Imaging, Inc., Burlington, VT, USA) in accordance with the manufacturer’s instructions and double checked by cytotechnologists. Reported cytological results were classified in accordance with the 2001 Bethesda system (Solomon et al., 2002).

### Colposcopy test

Colposcopy referral criteria were those of the most recent American Society of Colposcopy and Cervical Pathology (ASCCP) colposcopy standards (Wright, 2017), and colposcopy was performed following standard procedures. According to the ASCCP criteria and terminology, colposcopy impressions can be classified as benign, low-grade features, high-grade features, or cancer, as defined by the International Federation of Cervical Pathology and Colposcopy (Bornstein et al., 2012; Board, 2020; Höhn et al., 2021). In this study we categorized two groups for analyses: lower than low-grade squamous intraepithelial lesions (≤ LSIL), including benign and low-grade features, and higher than high-grade squamous intraepithelial lesions (≥ HSIL), including high-grade features and cancer.

### Statistical analyses

IBM SPSS Statistics version 28.0 (IBM Corporation, Armonk, NY, USA) software was used for statistical analyses. Frequency data are described as a percentage of cases, and co-occurrence of HPV and other bacteria was compared using chi-squared and Fisher’s exact probability tests, as appropriate. The Bonferroni correction test level was 0.05/3 = 0.0168 for multiple comparisons between different age groups. Univariate logistic regression was used to analyze the relative risk (odds ratio [OR] and 95% confidence interval [CI]) of HPV infection with different pathogens. After adjusting for age and AV/DIV infection status, the adjusted OR (OR_adj_) and 95% CI were calculated. All tests were two-sided, and a *p*-value < 0.05 was considered statistically significant.

## Results

### Clinical characteristics and prevalence of HPV and STIs

**Figure 2:**
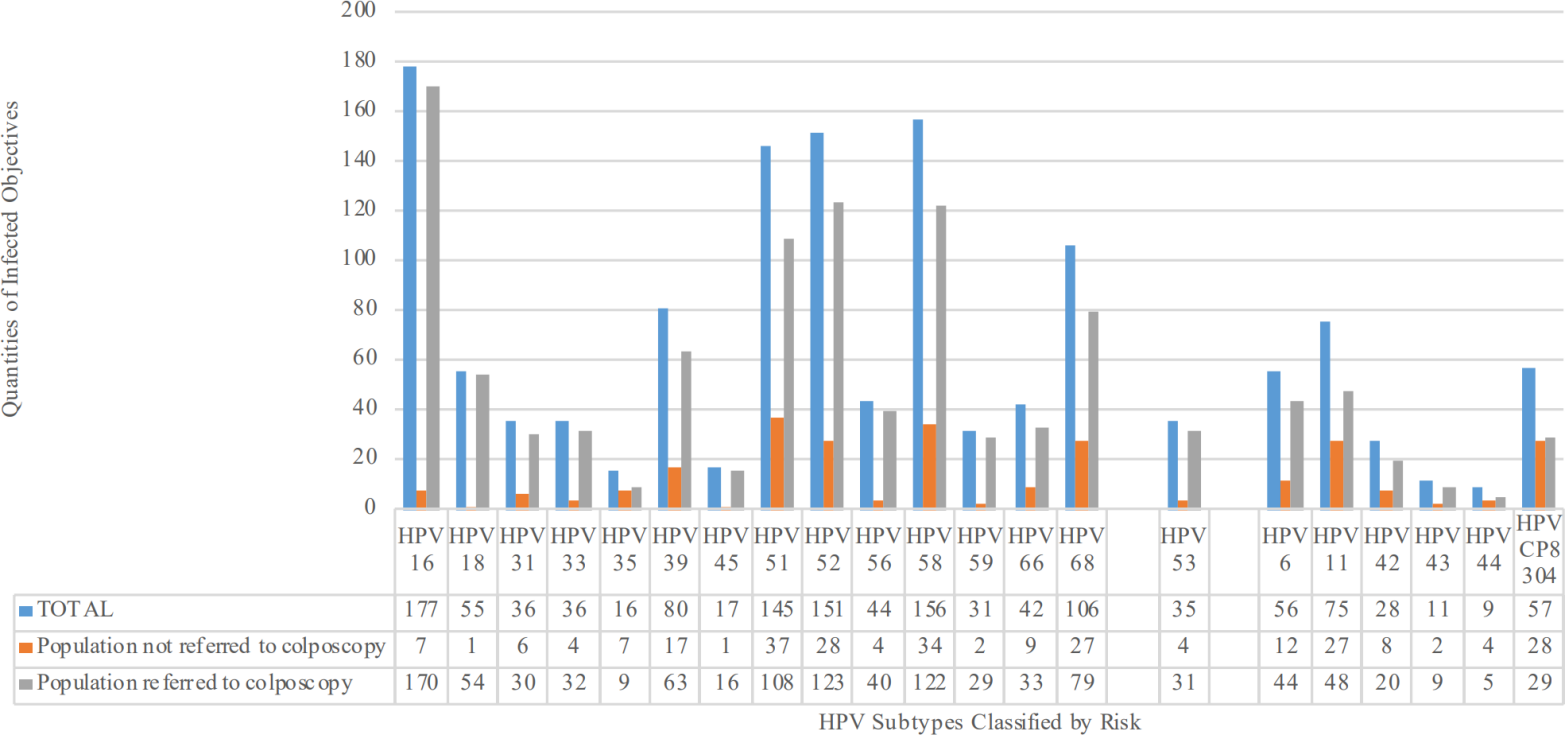
Number of people infected by different HPV subtypes classified by risk. HR-HPV subtypes include HPV16, 18, 31, 33, 35, 39, 45, 51, 52, 56, 58, 59, 66, and 68. Suspected HR-HPV subtypes include HPV53. LR-HPV subtypes include HPV6, 11, 42, 43, 44, and CP8304.

A total of 1,175 subjects screened at Peking University First Hospital from March 2021 to February 2022 were enrolled. The age range of the study cohort was 19–50 years, with an average ( ± SD) age of 35.37 ± 6.773 years, and an approximately normal distribution. The overall HPV infection rate was 66.0% (775/1,175), with a monotypic infection rate of 54.2% (420/775) and a polytypic infection rate of 45.8% (355/775). The HR-HPV infection rate in the HPV-positive group was 95.7% (742/775), of which HPV16/18 infection accounted for 29.2% (217/742). Calculating the number of infections by HPV subtype revealed that the three most common HR-HPV subtypes were HPV16, HPV58, and HPV52, whereas the three most common low-grade HPV subtypes were HPV11, HPV CP8304, and HPV6 (**Figure 2**). For TCT, 97.4% (1,145/1,175) were ≤ LSIL, and 2.6% (30/1,175) were ≥ HSIL. A total of 63.7% of the cohort (749/1,175) underwent colposcopy and cervical biopsy in accordance with the most recent ASCCP colposcopy standards (Wright, 2017), among whom 82.9% (621/749) were ≤ LSIL and 17.1% (128/749) were ≥ HSIL.

**Figure 3:**
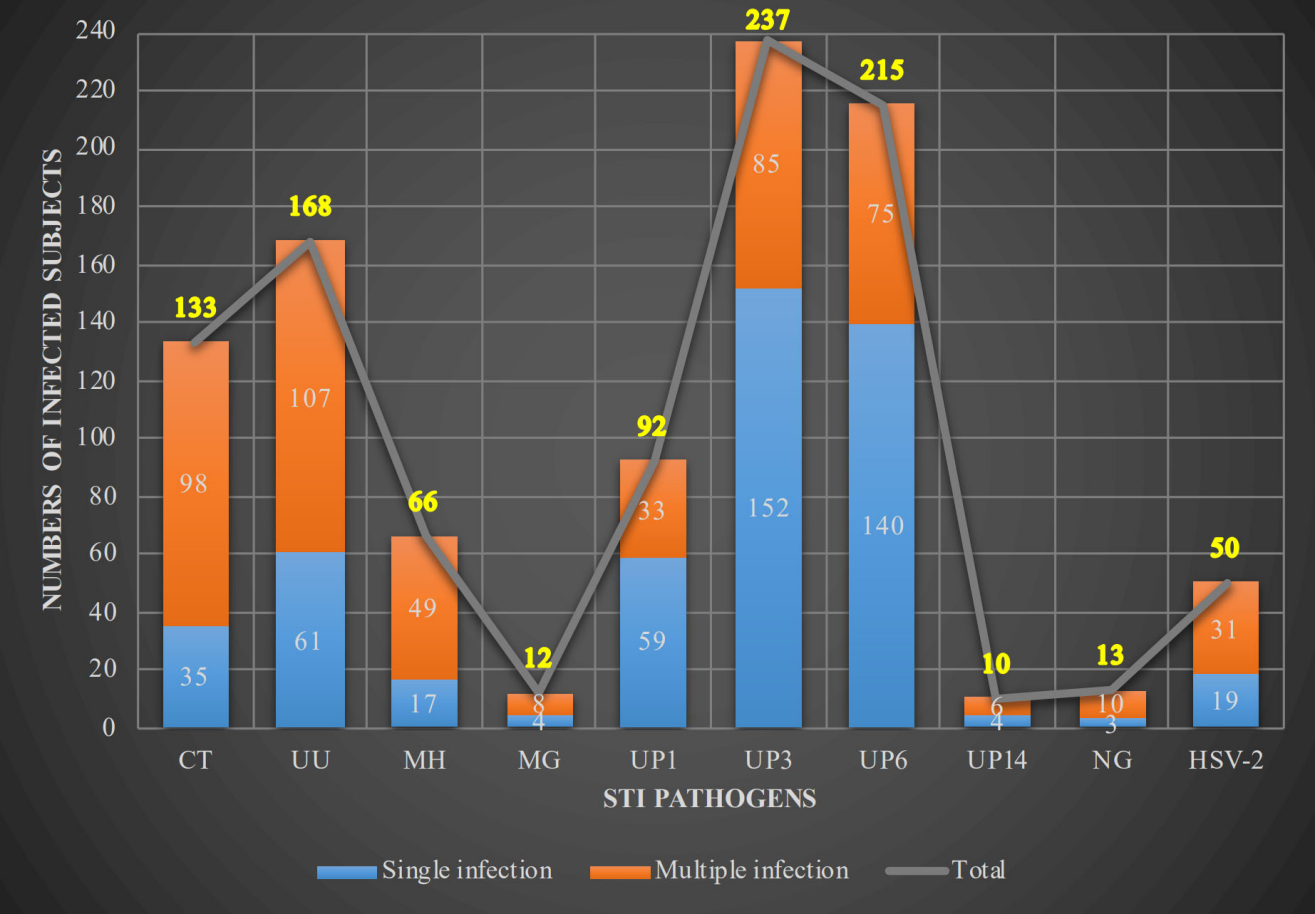
Types and proportions of the detected STI pathogens. Numbers of subjects with single infection, multiple infection, and total infection with detected STI pathogens shown. STI, sexually transmitted infection; CT, *Chlamydia trachomatis*; UU, *Ureaplasma urealyticum*; MH, *Mycoplasma hominis*; MG, *Mycoplasma genitalium*; UP, *Ureaplasma parvum*; NG, *Neisseria gonorrhoeae*; HSV-2, herpes simplex virus type 2.

The overall STI pathogen rate was 61.0% (717/1,175), of which single and multiple infections accounted for 68.9% (494/717) and 31.1% (223/717), respectively. The most prevalent infections were UP3 infection, at 20.2% (237/1,175), followed by UP6 infection, at 18.3% (215/1,175), UU infection, at 14.3% (168/1,175), and CT infection, at 11.3% (133/1,175) (**Figure 3**). We also tested for common types of vaginitis, including BV, AV/DIV, TV, and VVC, which were detected in 8.1% (95/1,175), 10.8% (127/1,175), 0.1% (1/1,175), and 3.1% (36/1,175) of subjects, respectively.

### Vaginal microbiome based on HPV infection status and TCT and biopsy results

There were no significant differences in BV, TV, or VVC infection rate between the groups with and without HPV infection; however, the AV/DIV infection rate was significantly higher in the HPV-positive group than in the HPV-negative group (*p* = 0.026, **Supplementary Table 1**). In the HPV-positive group, there were no significant differences in BV, AV/DIV, TV, or VVC infection rates between those with and those without HR-HPV infection (**Supplementary Table 2**). Likewise, in the HR-HPV-positive group, there were no significant differences in BV, AV/DIV, TV, or VVC infection rates between the groups with and without HPV16/18 (**Supplementary Table 3**). Moreover, when classified by single or multiple HPV infection type, there were no significant between-group differences in BV, AV/DIV, TV, or VVC infection rates (**Supplementary Table 4**). After adjusting for HPV infection status, there were no significant differences in BV, AV/DIV, TV, or VVC infection rate between groups based on TCT results (**Supplementary Table 5**). Those referred for colposcopy had a significantly higher AV/DIV infection rate than those not referred for colposcopy (*p* = 0.011, **Supplementary Table 6**), whereas there were no between-group differences in BV, AV/DIV, TV, or VVC infection rates between these groups (**Supplementary Table 7**).

### STIs in HPV infection status groups

Compared with the HPV-negative group, the overall STI pathogen rate, single STI pathogen rate, and multiple STI pathogens rate were all significantly higher (*p *< 0.001) in the HPV-positive group. Considering that there were > 50 potential STI pathogen combinations in the multiple STI group, and that that were < 15 cases of any combination, this group was combined for analyses. Although there was no age group effect for HPV infection rate (inconsistent with many previous studies), logistic regression was performed after adjusting for age and AV/DIV infection status. The UP6 (OR_adj_ 1.810, 95% CI 1.211–2.705; *p* = 0.004; **Table 1**) and HSV-2 (OR_adj_ 11.032, 95% CI 1.465–83.056; *p* = 0.020; **Table 1**) infection rates were significantly higher in the HPV-positive group than in the HPV-negative group. There were no other between-HPV infection group differences in STI pathogen rates.

**Table 1:** Infection rates of STIs in groups with or without HPV infection.

		Single STI										Multiple STIs	STI negative
		CT	UU	MH	MG	UP1	UP3	UP6	UP14	NG	HSV-2		
***n***		35	61	17	4	59	152	140	4	3	19	223	458
**HPV negative**, *n* (%)		9 (2.5)	19 (4.8)	7 (1.8)	0 (0.0)	25 (6.3)	52 (13.0)	37 (9.3)	2 (0.5)	1 (0.3)	1 (0.3)	45 (11.3)	202 (50.5)
**HPV positive**, *n* (%)		26 (3.4)	42 (5.4)	10 (1.3)	4 (0.5)	34 (4.4)	100 (12.9)	103 (13.3)	2 (0.3)	2 (0.3)	18 (2.3)	178 (23.0)	256 (33.0)
**Crude**	OR	1.751	1.338	0.847	–	0.797	1.172	1.792	0.593	1.190	11.005	3.121	2.068
	95% CI	0.811–3.780	0.766–2.340	0.319–2.245	–	0.467–1.360	0.814–1.688	1.200–2.677	0.083–4.230	0.108–13.170	1.463–82.796	2.144–4.543	1.616–2.647
	*p*	0.154	0.307	0.738	0.999^*^	0.405	0.392	0.004	0.602	0.887	0.020	< 0.001	< 0.001
**Adjusted**	OR	1.637	1.289	0.672	–	0.803	1.194	1.810	0.627	1.138	11.032	3.072	2.069
	95% CI	0.754–3.554	0.735–2.260	0.243–1.854	–	0.470–1.371	0.829–1.721	1.211–2.705	0.088–4.477	0.102–12.699	1.465–83.056	2.104–4.487	1.617–2.648
	*p*	0.212	0.375	0.443	0.999^*^	0.422	0.341	0.004	0.642	0.917	0.020	< 0.001	< 0.001

Percentages are row percentages with respect to the corresponding group with different HPV infection status.

^*^Fisher’s exact test.

Adjusted: Age, AV/DIV infection status.

STI, sexually transmitted infection; HPV, human papillomavirus; OR, odds ratio; CI, confidence interval; CT, *Chlamydia trachomatis*; UU, *Ureaplasma urealyticum*; MH, *Mycoplasma hominis*; MG, *Mycoplasma genitalium*; UP, *Ureaplasma parvum*; NG, *Neisseria gonorrhoeae*; HSV-2, herpes simplex virus type 2. -, Meaningless calculation results.

As HR-HPV is a persistent infection associated with cervical cancer, we further analyzed the STI status in groups with or without HR-HPV infection in the HPV-positive group. The UP6 infection rate was significantly higher in the HR-HPV-negative group than in the HR-HPV-positive group (OR_adj_ 0.373, 95% CI 0.153–0.909; *p* = 0.030; **Table 2**), whereas there were no differences in the other STI pathogen rates between the HR-HPV infection status groups. Because the most commonly reported HR-HPV infection types are HPV16 and 18, the STI status in groups with or without HPV16/18 infections in the HR-HPV-positive group was also analyzed. There were no between- HPV16/18 infection group differences in any STI pathogen rate (**Table 3**).

**Table 2:** Infection rates of STIs in groups with or without HR-HPV infection among HPV-positive subjects.

		Single STI										Multiple STIs	STI negative
		CT	UU	MH	MG	UP1	UP3	UP6	UP14	NG	HSV-2		
***n***		26	42	10	4	34	100	103	2	2	18	178	256
**HR-HPV negative**, *n* (%)		2 (6.1)	2 (6.1)	0 (0.0)	0 (0.0)	0 (0.0)	4 (12.1)	8 (24.2)	0 (0.0)	0 (0.0)	0 (0.0)	10 (30.3)	7 (21.2)
**HR-HPV positive**, *n* (%)		24 (3.2)	40 (5.4)	10 (1.3)	4 (0.5)	34 (4.6)	96 (12.9)	95 (12.8)	2 (0.3)	2 (0.3)	18 (2.4)	168 (22.6)	249 (33.6)
**Crude**	OR	0.458	0.787	–	–	–	0.954	0.372	–	–	–	0.472	0.533
	95% CI	0.102–2.068	0.178–3.474	–	–	–	0.317–2.867	0.153–0.902	–	–	–	0.176–1.265	0.228–1.245
	*p*	0.310	0.751	0.999^*^	0.999^*^	0.998^*^	0.933	0.029	0.999^*^	0.999^*^	0.998^*^	0.136	0.146
**Adjusted**	OR	0.434	0.727	–	–	–	0.981	0.373	–	–	–	0.423	0.514
	95% CI	0.094–2.008	0.163–3.244	–	–	–	0.324–2.970	0.153–0.909	–	–	–	0.157–1.136	0.219–1.204
	*p*	0.285	0.676	0.999^*^	0.999^*^	0.998^*^	0.973	0.030	1.000^*^	1.000^*^	0.998^*^	0.088	0.125

Percentages are row percentages with respect to the corresponding group with different HPV infection status.

^*^Fisher’s exact test.

Adjusted: Age, AV/DIV infection status.

STI, sexually transmitted infection; HPV, human papillomavirus; OR, odds ratio; CI, confidence interval; CT, *Chlamydia trachomatis*; UU, *Ureaplasma urealyticum*; MH, *Mycoplasma hominis*; MG, *Mycoplasma genitalium*; UP, *Ureaplasma parvum*; NG, *Neisseria gonorrhoeae*; HSV-2, herpes simplex virus type 2. –, Meaningless calculation results.

**Table 3:** Infection rates of STIs in groups with or without HPV 16/18 infection among HR-HPV-positive subjects.

		Single STI										Multiple STIs	STI negative
		CT	UU	MH	MG	UP1	UP3	UP6	UP14	NG	HSV-2		
*n*		24	40	10	4	34	96	95	2	2	18	168	249
HPV 16/18 negative, *n* (%)		13 (2.5)	32 (6.1)	5 (1.0)	4 (0.8)	24 (4.6)	61 (11.6)	66 (12.6)	2 (0.4)	0 (0.0)	14 (2.7)	126 (24.0)	178 (33.9)
HPV 16/18 positive, *n* (%)		11 (5.1)	8 (3.7)	5 (2.3)	0 (0.0)	10 (4.6)	35 (16.1)	29 (13.4)	0 (0.0)	2 (0.9)	4 (1.8)	42 (19.4)	71 (32.7)
Crude	OR	1.992	0.549	2.318	–	0.947	1.385	1.002	–	–	0.643	0.836	1.055
	95% CI	0.874–4.538	0.248–1.218	0.662–8.110	–	0.443–2.025	0.875–2.194	0.621–1.616	–	–	0.209–1.983	0.536–1.304	0.754–1.476
	*p*	0.101	0.140	0.188	0.999^*^	0.888	0.165	0.993	0.999^*^	0.999^*^	0.442	0.429	0.756
Adjusted	OR	1.921	0.535	2.427	–	0.951	1.396	1.004	–	–	0.649	1.366	1.034
	95% CI	0.834–4.425	0.241–1.189	0.657–8.967	–	0.444–2.035	0.880–2.215	0.622–1.621	–	–	0.211–2.002	0.726–2.569	0.737–1.450
	*p*-value	0.125	0.125	0.184	0.999^*^	0.896	0.156	0.987	0.999^*^	0.999^*^	0.452	0.334	0.847

Percentages are row percentages with respect to the corresponding group with different HPV infection status.

^*^Fisher’s exact test.

Adjusted: Age, AV/DIV infection status.

STI, sexually transmitted infection; HPV, human papillomavirus; OR, odds ratio; CI, confidence interval; CT, *Chlamydia trachomatis*; UU, *Ureaplasma urealyticum*; MH, *Mycoplasma hominis*; MG, *Mycoplasma genitalium*; UP, *Ureaplasma parvum*; NG, *Neisseria gonorrhoeae*; HSV-2, herpes simplex virus type 2. –, Meaningless calculation results.

Based on the number of HPV infection types, the STI status in the groups with various HPV infection genotypes was analyzed further, including single and multiple HPV infection profiles. There were no between-HPV infection genotype differences in STI pathogen rates (**Table 4**).

**Table 4:** Infection rates of STIs in groups with monotypic and multiple-type infection of HPV among HPV-positive subjects.

		Single STI										Multiple STIs	STI negative
		CT	UU	MH	MG	UP1	UP3	UP6	UP14	NG	HSV-2		
*n*		26	42	10	4	34	100	103	2	2	18	178	256
HPV monotypic infection, *n* (%)		12 (2.9)	30 (7.1)	5 (1.2)	2 (0.5)	19 (4.5)	64 (15.2)	57 (13.6)	2 (0.5)	0 (0.0)	0 (0.0)	71 (16.9)	158 (37.6)
HPV polytypic infection, *n* (%)		14 (3.9)	12 (3.4)	5 (1.4)	2 (0.6)	15 (4.2)	36(10.1)	46 (13.0)	0 (0.0)	2 (0.6)	18 (5.1)	107 (30.1)	98 (27.6)
Crude	OR	1.680	0.541	1.416	1.411	1.118	0.756	1.167	–	–	–	2.430	1.581
	95% CI	0.763–3.698	0.271–1.078	0.405–4.943	0.197–10.082	0.557–2.246	0.484–1.180	0.760–1.790	–	–	–	1.642–3.595	1.166–2.146
	*p*	0.197	0.081	0.586	0.732	0.754	0.219	0.480	0.999^*^	0.999^*^	0.998^*^	< 0.001	0.003
Adjusted	OR	1.761	0.540	1.750	1.327	1.123	0.747	1.161	–	–	–	2.413	1.596
	95% CI	0.794–3.906	0.270–1.079	0.476–6.431	0.184–9.543	0.559–2.258	0.478–1.168	0.756–1.782	–	–	–	1.626–3.583	1.175–2.168
	*p*	0.164	0.081	0.399	0.779	0.744	0.201	0.493	0.999^*^	0.999^*^	0.998^*^	< 0.001	0.003

Percentages are row percentages with respect to the corresponding group with different HPV infection status.

^*^Fisher’s exact test.

Adjusted: Age, AV/DIV infection status.

STI, sexually transmitted infection; HPV, human papillomavirus; OR, odds ratio; CI, confidence interval; CT, *Chlamydia trachomatis*; UU, *Ureaplasma urealyticum*; MH, *Mycoplasma hominis*; MG, *Mycoplasma genitalium*; UP, *Ureaplasma parvum*; NG, *Neisseria gonorrhoeae*; HSV-2, herpes simplex virus type 2. –, Meaningless calculation results.

### STIs in TCT status groups

Beyond the HPV–STI association, we explored whether or not STI may be associated with TCT results after adjustment for HPV infection status. Overall, there was not a between-TCT result group difference in STI pathogen rate after adjusting for HPV infection status. However, when analyzing only those with single infections, the STI infection rates for CT and MH were significantly higher in the group with TCT ≥ HSIL (*p* = 0.046 and *p* = 0.026, respectively; **Table 5**).

**Table 5:** Infection rates of STIs in groups with different TCT results.

		Single STI										Multiple STIs	STI negative
		CT	UU	MH	MG	UP1	UP3	UP6	UP14	NG	HSV-2		
*n*		35	61	17	4	59	152	140	4	3	19	223	458
TCT ≤** **LSIL, *n* (%)		32 (2.8)	59 (5.2)	15 (1.3)	4 (0.3)	59 (5.2)	148 (12.9)	137 (12.0)	4 (0.3)	3 (0.3)	19 (1.7)	217 (19.0)	448 (39.1)
TCT ≥** **HSIL, *n* (%)		3 (10.0)	2 (6.7)	2 (6.7)	0 (0.0)	0 (0.0)	4 (13.3)	3 (10.0)	0 (0.0)	0 (0.0)	0 (0.0)	6 (20.0)	10 (33.3)
Crude	OR	4.000	1.339	5.533	–	–	1.054	0.825	–	–	–	1.239	1.286
	95% CI	1.135–14.102	0.307–5.831	1.192–25.677	–	–	0.355–3.128	0.243–2.803	–	–	–	0.444–3.452	0.596–2.772
	*p*	0.031	0.697	0.029	0.999^*^	0.997^*^	0.924	0.758	0.999^*^	0.999^*^	0.998^*^	0.682	0.522
Adjusted	OR	3.643	1.265	5.867	–	–	1.020	0.736	–	–	–	0.971	1.099
	95% CI	1.026–12.942	0.289–5.533	1.241–27.745	–	–	0.343–3.036	0.216–2.513	–	–	–	0.343–2.746	0.506–2.388
	*p*	0.046	0.755	0.026	0.999^*^	0.997^*^	0.971	0.625	0.999^*^	0.999^*^	0.998^*^	0.955	0.812

Percentages are row percentages with respect to the corresponding group with different TCT results.

^*^Fisher’s exact test.

Adjusted: HPV infection.

TCT, liquid-based cytology test; STI, sexually transmitted infection; HPV, human papillomavirus; LSIL, low-grade squamous intraepithelial lesions; HSIL, high-grade squamous intraepithelial lesions; OR, odds ratio; CI, confidence interval; CT, *Chlamydia trachomatis*; UU, *Ureaplasma urealyticum*; MH, *Mycoplasma hominis*; MG, *Mycoplasma genitalium*; UP, *Ureaplasma parvum*; NG, *Neisseria gonorrhoeae*; HSV-2, herpes simplex virus type 2. –, Meaningless calculation results.

### STIs in biopsy status groups

Because the relation between HPV infection and cervical intraepithelial neoplasia (CIN) is well known, we also assessed whether STI pathogen status differed between groups with diverse cervical biopsy results after adjusting for HPV infection status among the 749 individuals referred for colposcopy. There was not a significant between-cervical biopsy group difference in either total STI pathogen rate or individual STI pathogen rates after adjusting for HPV infection status (**Table 6**).

**Table 6:** Infection rates of STIs in groups with different cervical biopsy results.

		Single STI										Multiple STIs	STI negative
		CT	UU	MH	MG	UP1	UP3	UP6	UP14	NG	HSV-2		
*n*		27	41	13	3	40	94	96	3	2	14	144	272
≤** **LSIL, *n* (%)		26 (4.2)	32 (5.2)	10 (1.6)	3 (0.5)	35 (5.6)	82 (13.2)	76 (12.2)	2 (0.3)	2 (0.3)	13 (2.1)	115 (18.5)	225 (36.2)
≥** **HSIL, *n* (%)		1 (0.8)	9 (7.0)	3 (2.3)	0 (0.0)	5 (3.9)	12 (9.4)	20 (15.6)	1 (0.8)	0 (0.0)	1 (0.8)	29 (22.7)	47 (36.7)
Crude	OR	0.188	1.481	1.550	–	0.716	0.713	1.432	2.571	–	0.387	1.207	0.979
	95% CI	0.025–1.405	0.684–3.209	0.419–5.736	–	0.273–1.875	0.373–1.364	0.828–2.477	0.231–28.636	–	0.050–2.992	0.722–2.019	0.660–1.453
	*p*	0.103	0.319	0.512	0.999^*^	0.496	0.307	0.199	0.442	0.999^*^	0.327	0.473	0.917
Adjusted	OR	0.177	1.535	1.743	–	0.831	0.649	1.315	3.546	–	–	1.095	0.898
	95% CI	0.024–1.324	0.699–3.371	0.454–6.685	–	0.312–2.212	0.338–1.247	0.756–2.286	0.272–46.308	–	0.042–2.531	0.650–1.845	0.601–1.341
	*p*	0.092	0.286	0.418	0.999^*^	0.710	0.194	0.332	0.334	0.999^*^	0.284	0.733	0.600

Percentages are row percentages with respect to the corresponding group with different biopsy results.

^*^Fisher’s exact test.

Adjusted: HPV infection.

STI, sexually transmitted infection; HPV, human papillomavirus; LSIL, low-grade squamous intraepithelial lesions; HSIL, high-grade squamous intraepithelial lesions; OR, odds ratio; CI, confidence interval; CT, *Chlamydia trachomatis*; UU, *Ureaplasma urealyticum*; MH, *Mycoplasma hominis*; MG, *Mycoplasma genitalium*; UP, *Ureaplasma parvum*; NG, *Neisseria gonorrhoeae*; HSV-2, herpes simplex virus type 2. –, Meaningless calculation results.

## Discussion

Increasing attention has been paid in recent years to possible HPV risk factors, including lower genital tract infection and vaginal microecological disorder, to prevent cervical lesions (Fracella et al., 2022; Guo et al., 2022). In this study we found that AV/DIV infection was higher in the HPV-positive group than in the HPV-negative group (*p* = 0.026), although no significant between-group differences in BV, TV, or VVC infection rates were observed. Previous investigators have shown a correlation between common vaginitis and HPV infection, suggesting that this is a risk factor (Wu et al., 2022; Huang et al., 2023). BV is an infectious disease caused by the displacement of vaginal *Lactobacillus* by potentially pathogenic microorganisms such as *Gardnerella vaginalis* and *Prevotella* species, resulting in vaginal microecology imbalance. A possible correlation between BV and HPV infection and cervical lesions has also been reported (Usyk et al., 2022; Martins et al., 2023). However, the relationship between TV, VVC, and HPV infection remain unclear (Wang et al., 2020; Heikal et al., 2023). AV/DIV, identified relatively late compared with other types of vaginitis, is characterized by *Lactobacillus* decline and increases in multiple aerobes, including *Streptococcus agalactiae* and *Streptococcus anginosus* (Donders et al., 2002). Previous studies have found that AV/DIV may be correlated with adverse pregnancy outcomes, such as abortion, stillbirth, premature delivery, and premature rupture of membranes, possibly due to toxin production or local effects on immunity, which in turn lead to infection (Han et al., 2019; Ma et al., 2022; Ncib et al., 2022).

Limited studies have previously been conducted to assess the association between AV/DIV and HPV infection. Jahic et al. (Jahic et al., 2013) found that AV/DIV may be associated with cervical LSIL. Vieira-Baptista et al. (Vieira-Baptista et al., 2016) found that moderate or severe AV/DIV, rather than BV, was independently associated with increased risk for major cervical cytological abnormalities. In addition, Plisko et al. (Plisko et al., 2021) found that moderate to severe AV/DIV and smoking were the most significant factors contributing to the development of CIN in HPV-positive women, especially high-grade CIN. Current speculation regarding pathogenesis includes that there is a link between AV/DIV, characterized by various degrees of inflammation and present with increased vaginal leucocytes, and highly increased concentrations of interleukin 1 beta (IL-1β) and IL-6, which are also characteristic of progressive CIN (Donders et al., 2017). Although additional large-sample studies will be needed to confirm and clarify this association, we suggest that greater attention is paid to AV/DIV in the association between vaginal microbiome and cervical lesions.

Recent studies have also identified a possible association between STIs and HPV infection. For instance, several studies have found that CT infection is associated with a high risk of, and persistent infection with, HPV (Naldini et al., 2019; Chen et al., 2020). Studies of the association between NG and HPV infection have been few. Others have found that persistent MH infection is associated with a high risk of HPV infection and CIN (Alotaibi et al., 2020; Klein et al., 2020). Similar correlations have been shown between UU and HPV infection (Kim et al., 2018). In this study, both single and multiple STI pathogen rates were found to be significantly higher in the HPV-positive group than in the HPV-negative group. The difference was particularly marked in the case of UP6 and HSV-2 infection rates (which refers to infection with only one of these organisms). However, by contrast, the UP6 infection rate was significantly higher in the HR-HPV-negative group than in the HR-HPV-positive group.

Detailed *Mycoplasma* typing has received insufficient clinical attention, partly because specific detection methods were lacking. In this study, we performed detailed *Mycoplasma* typing and, after analyzing the associations between common STI pathogens and HPV infection, were surprised to find significant differences in several UP subtype infection rates based on HPV infection status. To date, 14 *Ureaplasma* serovars have been identified, divided into two species: UP contains serovars 1, 3, 6, and 14, and UU contains the remaining 10 (Xiao et al., 2011). Few studies have addressed the association between UP and HPV. In 2016, Drago et al. (Drago et al., 2016) proposed that UP is a possible HPV-induced CIN enhancer agent, and confirmed this in a study 5 years later, showing that UP infection is a risk factor for persistent genital HPV infection (Ciccarese et al., 2021). In their study of 283 patients, Noma et al. (Noma et al., 2021) found that UP and HR-HPV coinfection increased LSIL risk. Similarly, a study of 480 patients found that UP14 and HR-HPV coinfection increased HSIL and cervical cancer risks, as did UP1 coinfection (Wang et al., 2019). A retrospective study of 668 patients found that UP6 infection was a risk factor for both HR-HPV infection and CIN, and that UP3 infection was a risk factor for CIN (Xie et al., 2021). Moreover, the possible role of UP in other illnesses has been investigated. Zanotta et al. found active UP3 infection in women with asymptomatic HR-HPV, and with idiopathic infertility, thus presenting a possible therapy target (Zanotta et al., 2019). Analogously, Rittenschober-Böhm et al. (Rittenschober-Böhm et al., 2019) observed that UP3 colonization increased the risk of spontaneous preterm birth and may be a target for therapeutic intervention studies. In this study, we found a significantly higher UP6 infection rate in the HPV-positive group than in the HPV-negative group (OR_adj_ 1.810, 95% CI 1.211–2.705; *p* = 0.004). Furthermore, the UP6 infection rate was significantly higher in the HR-HPV-negative group than in the HR-HPV-positive group (OR_adj_ 0.373, 95% CI 0.153–0.909; *p* = 0.030), suggesting that UP6 infection may be a risk factor for HPV infection. Our results are not uniformly consistent with those of previous studies, probably because of cohort differences or sample size limitations. Although the correlation between different UP serovars and HPV infection remains to be clearly established, based on our extant findings we nevertheless propose that further, detailed classification of *Mycoplasma*, especially different UP serovars, is needed to advance clinical practice.

Furthermore, the HPV infection rate group was significantly higher in the HSV-2-positive than in the HSV-2-negative group (OR_adj_ 11.032, 95% CI 1.465–83.056; *p* = 0.020). HSV-2 infection is among the most common STIs worldwide and is the leading cause of recurrent genital herpes. Many previous studies have used blood samples to detect HSV-2, partly because a specific kit for testing cervical samples was unavailable at the time. In this study, we used cervical samples to detect HSV-. Regarding the correlation between HSV-2 and HPV infection and cervical lesions, Smith et al. (Smith et al., 2002) found that HSV-2 seropositivity was associated with increased risks of squamous cell carcinoma (OR 2.19, 95% CI 1.41–3.40) and adeno- and adenosquamous cell carcinomas (OR 3.37, 95% CI 1.47–7.74), after adjustment for potential confounders. A cross-sectional study in 2020 observed significant differences in HSV-2 seroprevalence and HSV-2 active infection rates between negative and positive HR-HPV cases (Bahena-Román et al., 2020). Li et al. (Li and Wen, 2017) used eight datasets and a sample of 8,184 participants, finding that HSV-2 was associated with cervical cancer after adjusting for HR-HPV (OR_adj_ 1.90, 95% CI 1.09–3.34), suggesting that HSV-2 serostatus may serve as an independent predictor of cervical cancer. Our results appear to be consistent with these studies, though they are limited by the small sample size and the potential correlation between HSV-2 and HPV infection requires further study.

The main study limitation was its cross-sectional observational design. The sample size was also relatively small. Thus, larger, prospective studies are needed to confirm the relationship between STIs and HPV infection. Additional basic research is also needed to explain the specific underlying mechanisms.

In conclusion, HPV infection is associated with a disordered lower genital tract microecology, with a total STI pathogen rate of 61.0% in this cohort of participants who were undergoing routine cervical cancer screening. The total STI rate and multiple STI rate, as well as AV/DIV infection rate in HPV-positive patients were significantly higher than those in HPV-negative patients, which suggests that attention should be paid to the screening of lower genital tract infection in cervical cancer screening women, what’s more, especially the STI screening in HPV positive patients. Specifically, HSV-2 and *Mycoplasma*, especially UP6, infection rates were significantly higher in the HPV-positive group, emphasizing the importance of detailed typing and targeted treatment of *Mycoplasma* in clinical practice. Prospective cohort studies are now needed to further explore the relations and mechanisms between STI pathogens and HPV infection, and persistent infection and cervical lesions, to improve cervical cancer prevention, screening, diagnosis, and treatment.

## Data availability statement

The original contributions presented in the study are included in the article/**Supplementary Material.** Further inquiries can be directed to the corresponding author.

## Ethics statement

The studies involving human participants were reviewed and approved by The Ethics Committee of Peking University First Hospital (2021KY062). The patients/participants provided their written informed consent to participate in this study.

## Author contributions

BX conceived the study design. BX, DZ, and HB recruited volunteers and collected samples. DA and BX performed the data analysis. DA wrote the initial manuscript. BX and DA revised the manuscript. All authors contributed to the article and approved the submitted version.
