# Supplementary material for: Association between human papillomavirus infection and common sexually transmitted infections, and the clinical significance of different Mycoplasma subtypes
Source: Front Cell Infect Microbiol. 2023 Mar 16;13:1145215. doi: 10.3389/fcimb.2023.1145215 (PMC10061082; doi:10.3389/fcimb.2023.1145215)
Supplement: Supplementary file 1 [file DataSheet_1.pdf]

Supplementary Material

Association Between Common Sexually Transmitted Infection and Human Papillomavirus infection and Clinical Significance of Different Mycoplsma Subtypes

Disi A[1], Hui Bi[1], Dai Zhang[1], Bingbing Xiao[1*]

[1]Department of Obstetrics and Gynecology, Peking University First Hospital, Beijing, China

*** Correspondence:**

Prof. Bingbing Xiao: doctorxbb@163.com.

Supplementary TABLE 1 | Prevalence of HPV, STIs and common vaginitis in the study population

		**HPV negative [n (%)]**	**HPV positive [n (%)]**	**Total (n)**
Different infection status of STI	STI-	202 (50.5)	256 (33.0)	458
	STI+	198 (49.5)	519 (67.0)	717
	P	<0.001		
	STI-	202 (56.9)	256 (42.9)	458
	STI single infection	153 (43.1)	341 (57.1)	494
	P[#]	<0.001		
	STI-	202 (81.8)	256 (59.0)	458
	STI multiple infection	45 (18.2)	178 (41.0)	223
	P[#]	<0.001		
	STI single infection	153 (77.3)	341 (65.7)	494
	STI multiple infection	45 (22.7)	178 (34.3)	223
	P[#]	0.003		
Different infection status of BV	BV-	324 (34.1)	627 (65.9	951
	BV intermediate type	48 (37.2)	81 (62.8)	129
	BV+	28 (29.5)	67 (70.5)	95
	P	0.482		
Different infection status of AV/DIV	AV/DIV-	368 (35.1)	680 (64.9)	1048
	AV/DIV+	32 (25.2)	95 (74.8)	127
	P	0.026		
Different infection status of VVC	VVC-	391 (34.3)	748 (65.7)	1139
	VVC+	9 (25.0)	27 (75.0)	36
	P	0.245		
Different infection status of TV	TV-	400 (34.1)	774(65.9)	1174
	TV+	0 (0.0)	1 (100.0)	1
	P[*]	1.000		

[*] *Fisher's exact test*

[#] *P value of Bonferroni correlation: +<0.05/3=0.0168*

HPV, Human Papillomavirus; STIs, Sexually Transmitted Infections; BV, Bacterial Vaginosis; AV/DIV, Aerobic Vaginitis or Desquamative Inflammatory Vaginitis; TV, Vaginal Trichomoniasis; VVC, Vulvovaginal Candidiasis.

Supplementary TABLE 2 | Prevalence of common vaginitis in groups with different HR-HPV infection status

		HR-HPV negative [n (%)]	HR-HPV positive [n (%)]	Total (n)
Different infection status of BV	BV-	29 (87.9)	598 (80.6)	627
	BV intermediate type	4 (12.1)	77 (10.4)	81
	BV+	0 (0.0)	67 (9.0)	67
	P	0.149		
Different infection status of AV/DIV	AV/DIV-	31 (93.9)	649 (87.5)	680
	AV/DIV+	2 (6.1)	93 (12.5)	95
	P	0.280		
Different infection status of VVC	VVC-	33 (100.0)	715 (96.4)	748
	VVC+	0 (0.0)	27 (3.6)	27
	P*	0.998*		
Different infection status of TV	TV-	33 (100.0)	741 (99.9)	774
	TV+	0 (0.0)	1 (0.1)	1
	P*	1.000*		

* *Fisher's exact test*

HPV, Human Papillomavirus; STIs, Sexually Transmitted Infections; BV, Bacterial Vaginosis; AV/DIV, Aerobic Vaginitis or Desquamative Inflammatory Vaginitis; TV, Vaginal Trichomoniasis; VVC, Vulvovaginal Candidiasis.

Supplementary TABLE 3 | Prevalence of common vaginitis in groups with or without HPV16/18 infection status

		HPV16/18 negative [n (%)]	HPV16/18 positive [n (%)]	Total (n)
Different infection status of BV	BV-	426 (81.1)	172 (79.3)	598
	BV intermediate type	57 (10.9)	20 (9.2)	77
	BV+	42 (8.0)	25 (11.5)	67
	P	0.281		
Different infection status of AV/DIV	AV/DIV-	463 (88.2)	186 (85.7)	649
	AV/DIV+	62 (11.8)	31 (14.3)	93
	P	0.355		
Different infection status of VVC	VVC-	509 (97.0)	206 (94.9)	715
	VVC+	16 (3.0)	11 (5.1)	27
	P	0.186		
Different infection status of TV	TV-	525 (100.0)	216 (99.5)	741
	TV+	0 (0.0)	1 (0.5)	1
	P*	1.000*		

Supplementary TABLE 4 | Prevalence of common vaginitis in groups with single or multiple HPV infection

		**Single HPV [n (%)]**	**Multiple HPV [n (%)]**	**Total (n)**
Different infection status of BV	BV-	338 (80.5)	289 (81.4)	627
	BV intermediate type	46 (11.0)	35 (9.9)	81
	BV+	36 (8.6)	31 (8.7)	67
	P	0.861		
Different infection status of AV/DIV	AV/DIV-	366 (87.1)	314 (88.5)	680
	AV/DIV+	54 (12.9)	41 (11.5)	95
	P	0.580		
Different infection status of VVC	VVC-	410 (97.6)	338 (95.2)	748
	VVC+	10 (2.4)	17 (4.8)	27
	P	0.074		
Different infection status of TV	TV-	419 (99.8)	355 (100.0)	774
	TV+	1 (0.2)	0 (0.0)	1
	P*	1.000*		

Supplementary TABLE 5 | Prevalence of common vaginitis in groups with different TCT results

		≤ LSIL **[n (%)]**	≥ HSIL **[n (%)]**	**Total (n)**
Different infection status of BV	BV-	930 (81.2)	21 (70.0)	951
	BV intermediate type	125 (10.9)	4 (13.3)	129
	BV+	90 (7.9)	5 (16.7)	95
	P	0.077		
Different infection status of AV/DIV	AV/DIV-	1024 (89.4)	24 (80.0)	1048
	AV/DIV+	121 (10.6)	6 (20.0)	127
	P	0.108		
Different infection status of VVC	VVC-	1109 (96.9)	30 (100.0)	1139
	VVC+	36 (3.1)	0 (0.0)	36
	P*	0.998*		
Different infection status of TV	TV-	1144 (99.9)	30 (100.0)	1174
	TV+	1 (0.1)	0 (0.0)	1
	P*	1.000*		

Supplementary TABLE 6 | Prevalence of HPV, STIs and common vaginitis in the study population

**Characteristic**	***n***	**Prevalence [*n* (%)]**

		HPV+	STIs+	BV+	AV/DIV+	TV+	VVC+
Total	1175	775 (66.0)	717 (61.0)	95 (8.1)	127 (10.8)	1 (0.1)	36 (3.1)
Population not referred to colposcopy	426	133 (31.2)	240 (56.3)	35 (8.2)	33 (7.7)	0 (0.0)	15 (3.5)
Population referred to colposcopy	749	642 (85.7)	477 (63.7)	60 (8.0)	94 (12.6)	1 (0.1)	21 (2.8)
P		<0.001	0.013	0.863	0.011	1.000*	0.493

* *Fisher's exact test.*

HPV, Human Papillomavirus; STIs, Sexually Transmitted Infections; BV, Bacterial Vaginosis; AV/DIV, Aerobic Vaginitis or Desquamative Inflammatory Vaginitis; TV, Vaginal Trichomoniasis; VVC, Vulvovaginal Candidiasis

Supplementary TABLE 7 | Prevalence of common vaginitis in groups with different biopsy results

		**≤ LSIL [n (%)]**	**≥ HSIL [n (%)]**	**Total (n)**
Different infection status of BV	BV-	504 (81.2)	100 (78.1)	604
	BV intermediate type	69 (11.1)	16 (12.5)	85
	BV+	48 (7.7)	12 (9.4)	60
	P	0.422		
Different infection status of AV/DIV	AV/DIV-	546 (87.9)	109 (85.2)	655
	AV/DIV+	75 (12.1)	19 (14.8)	94
	P	0.391		
Different infection status of VVC	VVC-	603 (97.1)	125 (97.7)	728
	VVC+	18 (2.9)	3 (2.3)	21
	P	0.730		
Different infection status of TV	TV-	621 (100.0)	127 (99.2)	748
	TV+	0 (0.0)	1 (0.8)	1
	P*	1.000*		

* *Fisher's exact test*

HPV, Human Papillomavirus; LSIL, low-grade squamous intraepithelial lesions; HSIL, high-grade squamous intraepithelial lesions; BV, Bacterial Vaginosis; AV/DIV, Aerobic Vaginitis or Desquamative Inflammatory Vaginitis; TV, Vaginal Trichomoniasis; VVC, Vulvovaginal Candidiasis.

Supplementary TABLE 8 | Test results for each subject

Number	Age	Colposcopy（0-No；1-Yes）	HPV	TCT（0-Blank；1-NILM；2-ASCUS；3-ASC-H；4-LSIL；5-HSIL；6-SCC；7-AGC-NOS；8-AGC-FN；9-AIS；10-CA；11-Others）	Biopsy（No-0，≦LSIL-1，≧HSIL-2）	STI（Negative-----0，CT------1，UUU-------2，MH-------3，MG-------4，UP1------5，UP3------6，UP6------7，UP14-----8，NG-------9 ，HSV2-----10）	Nugent Score	AV Score	Vaginal PH（3.8-4.5---0，>4.5------1）	TV（Negative---0，Positive---1）	VVC（Negative---0，Positive---1）
11001	27	0	-	1	0	6	0	0	1	0	0
11002	22	0	-	1	0	2	5	2	0	0	0
11003	39	0	-	1	0	6	0	0	0	0	0
11007	27	0	-	1	0	0	0	0	0	0	0
11038	35	0	-	1	0	6	0	0	0	0	0
11075	27	0	11	1	0	1、2	1	0	0	0	0

			51，								
11092	27	0	CP8304	1	0	0	0	0	0	0	0
11008	47	0	-	1	0	6	0	0	0	0	1
11071	35	0	42	1	0	2	1	0	0	0	0
11091	35	0	-	1	0	0	0	0	0	0	0
11108	35	1	16	1	1	6	1	0	1	0	0
11012	47	0	-	1	0	1、3	3	2	1	0	0
11116	27	0	-	1	0	0	0	0	0	0	0
11120	35	0	52，58	1	0	7	1	0	0	0	0
11160	27	0	-	1	0	0	0	0	0	0	1
11016	23	0	-	1	0	2	7	2	1	0	1
11225	27	0		1	0	1	0			0	0
11150	35	0	51，68	1	0	6	1	0	0	0	1
11164	35	0	-	1	0	2	0	0	0	0	0
11172	35	0	-	1	0	0	3	2	0	0	0
11186	35	0	58	1	0	0	0	0	0	0	0
11278	27	1	68、42	4	1	2	0			0	0

11242	35	0		5	0	7	0			0	0
11250	35	0		1	0	0	0			0	0
11257	35	0		1	0	0	4			0	0
11264	35	1	16、6	1	1	7	8			0	0
11306	35	0	16	1	0	0	1			0	0
11301	27	0	39	1	0	6、7	0			0	0
11029	46	0	58	1	0	7	1	0	0	0	0
11306	35	0	16	1	0	0	1			0	0
11305	27	0		1	0	6	0			0	0
11353	35	0		1	0	0	0			0	0
11312	27	0		1	0	0	2			0	0
11399	35	0		1	0	5	0			0	0
11035	20	0	-	1	0	0	0	0	0	0	0
11417	35	1		1	1	1、6	0			0	0
11312	27	0		1	0	0	2			0	0
11455	35	0		1	0	0	1			0	0
11473	35	0		1	0	0	1			0	0

11040	19	0	-	1	0	0	5	4	0	0	0
11041	44	1	16，42	1	1	3	8	3	1	0	0
11042	43	1	16，11	1	1	1、7	8	3	0	0	0
11490	35	0		1	0	6	0			0	0
11044	45	0	11	1	0	6	3	2	0	0	0
11796	35	0		1	0	0	1			0	0
11799	35	0		1	0	7	8			0	0
11047	50	1	16，11	1	1	6	1	0	0	0	1
12001	35	1	-	4	2	0	0	0	1	0	0
12017	35	1	16，18	1	1	7、9	0	0	0	0	0
11050	47	0	51，11，44	1	0	0	0	0	0	0	0
12081	35	1	-	1	1	2、6、7	8	3	0	0	0
11052	49	0	-	1	0	0	3	2	0	0	0
12106	35	1	56，68	1	1	7	6	2	0	0	0
11319	27	0		1	0	6	0			0	0
11055	43	0	52	1	0	7	0	1	1	0	0

11056	43	0	51	1	0	0	1	0	1	0	0
11319	27	0		1	0	6	0			0	0
12123	35	1	52	1	1	0	1	0	0	0	0
11364	27	0		1	0	0	1			0	0
11790	27	0		1	0	1、6	1			0	0
11061	26	0	35，39	1	0	5	5	3	0	0	1
12157	35	1	58	2	2	6	1	0	0	0	0
11063	44	0	51	1	0	5	4	3	0	0	0
11064	45	1	16	1	1	0	0	0	0	0	0
11065	25	0	-	1	0	1、10	1	0	0	0	0
11066	23	1	16，51，52，11	1	2	7	1	0	0	0	1
12080	27	1	31，39，51，66，6	2	2	2、10	1	1	0	0	0
12161	35	1	56	1	1	2	0	0	0	0	0
12199	35	1	18	1	1	0	2	0	0	0	0
11070	24	0	-	1	0	1、2	1	0	0	0	0

H-015	35	1	59	1	1	7	0	0	0	0	0
H-018	35	1	18	1	1	1、2、7	0	0	0	0	0
12085	27	1	-	2	1	0	1	0	0	0	0
12109	27	1	31	2	1	0	0	0	0	0	0
12116	27	1	66	4	1	6	0	0	0	0	0
H-025	35	1	51	1	1	6	0	0	0	0	0
H-035	35	1	16、18、 12 other HR-HPV	4	2	5	0	0	0	0	0
H-004	27	1	16、58	2	2	6	1	2	0	0	0
11079	22	1	16，18	1	1	1	8	3	0	0	0
H-088	35	1	18	2	2	3	5	4	1	1	0
H-092	35	1	12 other HR-HPV	4	1	0	8	4	1	0	0
H-024	27	1	18	1	1	6	4	4	1	0	1
11083	49	0	11	1	0	1	1	0	0	0	0
H-097	35	1	39、51	3	1	7	1	0	0	0	0

H-106	35	1	16	1	2	6	0	1	0	0	0
11086	43	0	-	1	0	7、3	8	3	1	0	0
11087	43	0	-	1	0	0	0	0	0	0	0
H-147	35	1	16	1	1	2	2	3	1	0	0
H-155	35	1	12 other HR-HPV	2	2	6	0	0	0	0	0
H-027	27	1	39	4	1	5、6	4	2	0	0	1
H-161	35	1	52	5	2	0	8	3	0	0	0
H-047	27	1	16	2	2	0	0	0	0	0	0
H-058	27	1	58、6	1	1	7、3	7	2	0	0	0
H-162	35	1	16、56、52	2	2	7	0	1	0	0	0
11095	26	0	51，52，11，CP8304	1	0	2、5	0	0	0	0	0
H-181	35	1	18、45	1	1	0	2	3	1	0	0
H-068	27	1	51	2	1	7	0	0	0	0	0

H-139	27	1	33	1	2	0	1	0	0	0	0
H-221	35	1	52	1	1	7	2	2	0	0	1
H-247	35	1	52	1	1	0	0	0	0	0	0
11101	47	0	-	1	0	0	0	0	0	0	0
H-252	35	1	52	1	1	0	1	0	0	0	0
H-295	35	1	18	1	1	2、1	8	3	1	0	0
11068	36	0	-	1	0	0	8	3	0	0	0
11105	25	1	-	1	1	2、3	8	3	0	0	0
H-236	27	1	66	4	1	0	2	0	0	0	0
H-251	27	1	51	1	2	7	1	0	1	0	0
11104	36	0	11,CP8304,<br>39,68	1	0	0	4	4	1	0	0
11109	22	0	-	1	0	0	2	0	1	0	0
H-264	27	1	18	1	1	7、1	1	1	0	0	0
11111	44	0	-	1	0	7	1	0	0	0	0
11073	28	0	-	1	0	9、7、4、3	0	0	0	0	0

11113	43	0	39，52，58，CP8304	1	0	2	0	0	0	0	1
11114	49	1	18，31	1	1	7	0	0	0	0	0
11115	47	0	-	1	0	0	1	0	0	0	0
11090	28	0	51,11,CP8304	1	0	1、2、6	1	0	1	0	0
11124	28	0	51	1	0	0	0	2	0	0	0
11118	45	0	39,51,58,CP8304,11	1	0	1、2	0	0	0	0	0
11125	36	0	51,56,58,68,44	1	0	0	1	1	0	0	0
11152	36	0	-	1	0	0	4	2	1	0	1
11121	25	0	-	1	0	5	0	0	0	0	0
11122	21	0	-	1	0	1	0	0	0	0	0
11123	43	0	-	1	0	0	0	0	0	0	0
11147	28	0	-	1	0	6	0	0	1	0	0

11165	36	1	58，59	1	1	5	1	0	0	0	0
11126	23	0	-	1	0	7	0	0	0	0	0
11177	36	0	35，58	1	0	0	1	0	1	0	0
11128	25	0	-	1	0	7	1	0	0	0	0
11129	49	0	68，6	1	0	6、10	2	0	0	0	0
11130	48	0	45，51，52，58，68，	1	0	2、3、10	5	2	0	0	0
11207	28	0	51	1	0	2、3	8	3	0	0	0
11208	28	1	16	1	1	6	6	2	1	0	0
11133	47	1	16，68，6	1	1	9、10	8	3	0	0	0
11184	36	0	58，68	1	0	1、7、10	0	0	1	0	0
11226	36	1	16，58，59	1	1	7	0			0	0
11239	28	0		1	0	1、7	2			0	0
11281	28	0		1	0	6	2			0	0
11298	28	0		2	0	5、6	0			0	0

11141	45	0	-	1	0	0	0	0	0	0	0
11244	36	0		1	0	0	3			0	0
11326	28	1	16	1	1	6	0			0	0
11145	43	0	-	1	0	6	1	0	0	0	0
11254	36	0		1	0	6	8			0	0
11342	28	0	16、39	1	0	6、3	0			0	0
11148	22	0	-	1	0	6、10	1	0	0	0	0
11263	36	0		1	0	2	0			0	0
11274	36	0		1	0	7、3	8			0	0
11276	36	0		1	0	0	0			0	0
11153	26	0	-	1	0	7	0	0	0	0	0
11154	24	0	58，68，6，11	1	0	0	1	0	0	0	0
11155	43	0	52,58,68,6,CP8304	1	0	10	8	3	0	0	0
11158	45	0	-	1	0	0	0	0	0	0	0
11284	36	0		1	0	5	0			0	0

11360	28	0	16	2	0	2	0			0	0
11310	36	0		1	0	6	0			0	0
11310	36	0		1	0	6	0			0	0
11163	25	0	-	1	0	0	1	0	0	0	0
11335	36	0		1	0	6	0			0	0
11352	36	0		1	0	0	2			0	1
11166	25	0	-	1	0	1、2	2	3	1	0	0
11384	28	0		1	0	6	0			0	0
11406	28	0		1	0	5、6	1			0	0
11380	36	0		1	0	1	1			0	0
11420	28	0		1	0	0	6			0	0
11439	28	1	58	1	1	0	0			0	0
11448	28	0		1	0	0	1			0	0
11381	36	0		1	0	0	1			0	0
11390	36	0		1	0	0	1			0	0
11179	26	1	-	4	1	0	0	0	0	0	0
11180	49	0	CP8304	1	0	2、6	1	0	0	0	0

11400	36	1	58	3	1	0	0			0	0
11182	22	0	51，58，68	1	0	6	0	0	0	0	0
11409	36	0	52、58	1	0	1、6	1			0	0
11449	36	0		1	0	0	0			0	0
11467	36	0	52	1	0	0	0			0	0
11792	36	0		1	0	0	1			0	0
11451	28	0		1	0	0	0			0	0
11190	44	0	6	1	0	7	3	3	0	0	0
11464	28	0		1	0	7	0			0	0
11794	36	0		1	0	10	0			0	0
12034	28	1	39，52，68，53，11	1	1	1、2、6	1	0	0	0	0
12069	28	1	31,52,53,42,43	1	2	2、7	0	0	0	0	0

			66,6,44,CP								
11209	25	0	8304	1	0	1、2、5	0	0	0	0	0
11795	36	0		1	0	0	0			0	0
12098	28	1	58	4	2	1、7	1	0	0	0	0
12133	28	1	33，52，59	2	1	7	0	0	0	0	0
12007	36	1	16.39.52.56	3	1	0	4	4	1	0	0
12136	28	1	66	1	1	1	8	3	0	0	0
12139	28	1	52	2	1	7	0	0	0	0	0
12166	36	1	52	2	1	0	5	3	0	0	0
12146	28	1	56,11	4	1	0	2	1	0	0	0
11218	45	0	-	1	0	6	6	2	0	0	0
12151	28	1	52	1	1	0	0	0	0	0	0
12173	36	1	-	1	1	5	1	0	0	0	0
12174	36	1	68	1	1	6	2	0	0	0	0
12180	28	1	-	1	1	6	0	0	0	0	0
12189	28	1	58	1	2	7	0	0	0	0	0

12190	28	1	-	1	1	0	0	0	0	0	0
12175	36	1	58,59	1	1	5	1	0	0	0	0
12216	28	1	52	1	1	0	5	3	0	0	0
H-012	36	1	68	2	1	5	0	0	0	0	0
H-013	36	1	16、31	1	1	1	0	0	0	0	0
H-046	36	1	33	1	1	0	0	0	0	0	0
12217	28	1	58	4	1	2、7	0	0	0	0	0
12230	28	1	16，52	1	1	6	1	0	1	0	0
H-029	28	1	51	1	1	0	0	1	0	0	0
11235	46	0	16，33，42	1	0	2、6、3	8			0	0
H-079	36	1	33、52、58	1	1	0	0	0	0	0	0
H-032	28	1	16	1	1	5	0	0	0	0	0
11238	43	0		1	0	7	8			0	0
H-038	28	1	16	1	1	7	0	0	0	0	0
H-082	36	1	16、51	2	1	6	1	0	1	0	0

H-109	36	1	18、12 other HR-HPV	1	1	6	1	0	0	0	0
H-045	28	1	66、33、43	1	2	6	0	0	0	0	0
H-131	36	1	39	1	1	5	0	0	0	0	0
11245	26	0		1	0	6	0			0	0
H-050	28	1	51	4	1	0	1	0	0	0	0
H-144	36	1	16	1	1	5	0	1	1	0	0
H-178	36	1	12 other HR-HPV	4	1	0	1	0	0	0	0
H-180	36	1	52	1	1	7	0	0	0	0	0
11253	48	0		1	0	0	4			0	0
H-290	36	1	16	1	1	1	0	0	0	0	0
H-073	28	1	16	1	1	2	1	0	0	0	0
H-220	28	1	18	1	1	1	4	2	0	0	1
11021	37	0	-	1	0	0	2	2	0	0	0

H-235	28	1	16、52、68	1	2	0	0	1	0	0	0
H-257	28	1	16、18	2	1	7、1	1	0	0	0	0
H-276	28	1	16、39、82	2	2	7	1	0	0	0	0
11262	49	0		1	0	6、7	0			0	0
11026	37	0	-	1	0	0	1	0	0	0	0
11027	37	0	52	1	0	6	0	0	0	0	0
11036	37	0	-	1	0	9	0	0	0	0	0
11046	37	0	39，51，52，11	1	0	0	1	0	0	0	0
11048	37	0	51	1	0	0	1	0	0	0	0
11097	29	0	52，11，CP8304	1	0	1、2	5	3	1	0	0
11140	29	0	51，68，6	1	0	10	1	0	0	0	0
11171	29	0	-	1	0	6	0	0	0	0	0
11053	37	1	11	1	1	0	1	0	1	0	0

11058	37	1	51	1	1	6	1	0	0	0	0
11081	37	0	-	1	0	1、2、6	1	0	0	0	0
11211	29	0	-	1	0	0	0	0	0	0	0
11280	25	0		1	0	0	4			0	0
11261	29	0		1	0	6、7	1			0	0
11282	45	0		1	0	0	0			0	0
11283	24	0		1	0	7	0			0	0
11100	37	0	-	1	0	0	4	2	0	0	0
11119	37	0	42	1	0	1、2、6	0	0	0	0	0
11222	37	0		1	0	0	0			0	0
11287	44	0		1	0	7	1			0	0
11289	25	0		1	0	2	0			0	0
11304	29	1		4	1	0	4			0	0
11292	26	0		1	0	0	0			0	0
11267	37	0		1	0	0	3			0	0
11294	24	0		1	0	6	0			0	0
11395	29	0		1	0	7	0			0	0

11428	29	0		1	0	0	1			0	1
11458	29	0		1	0	6	0			0	0
11285	37	0	36、52	1	0	0	1			0	0
12066	29	1	39,52,68,6	1	2	5、10	1	0	0	0	0
12091	29	1	51,52,58,6	1	1	0	0	0	0	0	0
12169	29	1	52	1	1	7	0	0	0	0	0
12182	29	1	58	2	2	0	0	0	0	0	0
H-044	29	1	16	1	2	6	2	2	0	0	0
H-048	29	1	18	2	1	5	2	0	0	0	0
11308	37	0		1	0	6	5			0	0
11308	37	0		1	0	6	5			0	0
H-100	29	1	58	1	1	7	4	2	0	0	0
H-115	29	1	16	1	2	6	2	0	0	0	0
11371	37	1		2	1	6	4			0	0
11372	37	0		1	0	0	7			0	0
11393	37	0	31	1	0	0	0			0	0
11432	37	0		1	0	3	8			0	0

11469	37	0		1	0	6	1			0	0
12053	37	1	58.68.6	1	1	0	3	2	0	0	0
H-132	29	1	16	1	1	6	1	0	0	0	0
H-154	29	1	18、33	4	2	5	1	0	0	0	0
H-224	29	1	16	1	1	6、3	3	3	0	0	1
H-246	29	1	31、51	2	1	2	1	0	0	0	0
H-248	29	1	12 other HR-HPV	2	1	7	1	0	0	0	0
H-297	29	1	16	1	1	0	1	0	0	0	0
11315	43	0		1	0	6	8			0	0
11315	43	0		1	0	6	8			0	0
11015	30	0	53,43	1	0	2、5	1	0	0	0	1
11017	30	0	-	1	0	7	1	0	0	0	0
11074	30	0	51	1	0	4	0	1	0	0	0
11082	30	0	-	1	0	0	1	0	0	0	0
12075	37	1	52，68，66，6	1	2	0	4	2	0	0	0

12093	37	1	58	5	2	0	3	2	0	0	0
11093	30	0	-	1	0	5	0	0	0	0	0
11144	30	1	16，68，6，11	1	1	10	1	0	0	0	0
11320	43	0		1	0	6	0			0	0
12143	37	1	58	4	2	7	1	0	0	0	0
11323	45	0	53	1	0	2、6	7			0	0
11174	30	0	58	1	0	5、6、7、8	3	2	0	0	0
11175	30	1	-	1	1	5	1	0	0	0	0
12171	37	1	-	1	1	5	1	0	0	0	0
H-081	37	1	12 other HR-HPV	4	2	7	0	0	0	0	0
11333	45	0		1	0	0	0			0	0
11191	30	0	-	1	0	5	0	0	0	0	0
H-085	37	1	16、58	1	2	2、4	0	0	0	0	0
11227	30	0		1	0	0	0			0	0
H-090	37	1	58	4	2	0	1	2	0	0	1

11346	26	0		2	0	0	1			0	0
H-104	37	1	16、51	1	1	6	0	2	0	0	0
11237	30	0		1	0	0	4			0	0
H-111	37	1	16	1	2	7	2	2	0	0	0
H-113	37	1	16、52	1	1	0	4	2	0	0	0
H-141	37	1	16、45	1	1	0	10	3	1	0	0
H-192	37	1	56、58	1	1	6、3	7	4	1	0	1
11246	30	0		1	0	0	1			0	0
H-194	37	1	18	1	1	7	0	0	0	0	0
H-198	37	1	16	1	1	5	0	1	0	0	0
H-211	37	1	42、58	1	2	0	0	0	0	0	0
11255	30	0		1	0	6	1			0	0
11291	30	0		1	0	0	0			0	0
11365	23	0		1	0	7	0			0	0
H-214	37	1	12 other HR-HPV	2	1	6	0	0	0	0	0
11356	30	0		1	0	0	3			0	0

11368	43	0		1	0	0	1			0	0
H-245	37	1	39	2	1	2	0	0	0	0	0
11404	30	0		1	0	0	0			0	0
H-268	37	1	33	2	2	7	0	0	0	0	0
H-280	37	1	58	1	1	0	0	0	0	0	0
H-291	37	1	16	2	1	0	8	2	1	0	0
11374	43	1		1	1	0	0			0	0
11375	43	1		1	1	7	0			0	0
11416	30	0		1	0	0	1			0	0
11457	30	0		1	0	0	0			0	0
H-293	37	1	16	1	1	0	1	0	0	0	0
11023	38	0	-	1	0	0	0	0	0	0	0
11024	38	0	-	1	0	0	1	0	0	0	0
11039	38	0	-	1	0	0	0	2	0	0	0
12004	30	1	56	3	1	6	0	0	0	0	0
11045	38	0	-	1	0	0	1	0	0	0	1
11049	38	0	51，11	1	0	0	1	0	0	0	0

11387	43	0		1	0	0	0			0	0
12090	30	1	31,35,58	2	1	2、6、8、10	1	0	0	0	0
11389	25	0	52	1	0	2	3			0	0
11072	38	0	-	1	0	1、2	1	0	0	0	0
11391	44	0		1	0	0	5			0	0
11392	19	0	16	1	0	0	0			0	0
11077	38	0	-	1	0	3	8	3	0	0	0
11394	24	0		1	0	1、2	1			0	0
12096	30	1	58,68	1	1	1、6、10	0	0	0	0	0
11396	47	0		1	0	6	4			0	0
11397	46	0		1	0	0	3			0	0
11088	38	0	51，11	1	0	1、7	1	0	0	0	0
11103	38	0	-	1	0	7	4	4	0	0	0
11143	38	0	-	1	0	0	5	5	1	0	0
12108	30	1	56，53	1	1	7	1	0	0	0	0
12130	30	1	16,68,6,11	1	1	6、10	1	0	0	0	0

			68,6,CP830								
11146	38	0	4	1	0	10	0	0	0	0	0
11183	38	0	-	1	0	6	1	0	0	0	0
11187	38	1	52	1	1	1	0	0	0	0	0
11247	38	0		1	0	3	8			0	0
11268	38	0		1	0	6	6			0	0
11277	38	0		2	0	0	0			0	0
12135	30	1	59	2	2	2	4	4	0	0	0
11286	38	0		1	0	0	0			0	0
11418	23	0	42	1	0	1、6	1			0	0
11321	38	0		2	0	7	0			0	0
12164	30	1	33	4	1	7	0	0	0	0	0
11331	38	0		1	0	0	0			0	0
11422	24	0		1	0	5、7	0			0	0
11423	23	0	52	1	0	6	8			0	0
11359	38	1	51	1	2	0	0			0	0
12167	30	1	53	1	1	7	2	0	0	0	0

11426	25	0		1	0	7	1			0	0
12178	30	1	52	1	1	6	0	0	0	0	0
H-020	30	1	52、39、59	1	1	1、7	1	0	0	0	1
11379	38	0		1	0	0	0			0	0
11386	38	0		1	0	0	1			0	0
11408	38	0		1	0	7	1			0	0
11434	21	0		1	0	0	0			0	0
H-065	30	1	16	1	1	7	0	0	0	0	0
11424	38	0		1	0	0	0			0	0
11440	38	1	52、68	1	1	6	8			0	0
11442	38	0	11	1	0	6	3			0	0
H-078	30	1	16	1	1	0	0	0	0	0	0
11446	38	1	CP8304	1	1	7	0			0	0
11468	38	0		1	0	0	8			0	0
11443	45	0		2	0	0	0			0	0
11475	38	1		1	1	0	4			0	0

11797	38	0		1	0	3	8			0	0
12005	38	1	51	1	2	3、6	0	0	0	0	0
H-118	30	1	16	1	2	6、7	1	0	0	0	0
12014	38	1	51	2	1	0	0	0	0	0	0
11450	22	0		1	0	0	8			0	0
H-119	30	1	58	2	1	6	1	0	0	0	0
11453	43	0		1	0	2	8			0	0
11454	48	0		1	0	0	0			0	0
12015	38	1	-	1	1	0	0	2	0	0	0
H-158	30	1	31	1	2	2、5	8	3	0	0	0
H-165	30	1	52	1	2	0	0	0	0	0	0
11460	49	1		1	1	7	0			0	0
11462	45	1		1	1	6	8			0	0
12016	38	1	16.51.52.53<br>.11	4	1	0	0	0	0	0	0
H-166	30	1	33、58	1	1	7	0	0	0	0	0
11465	45	0		1	0	0	0			0	0

12032	38	1	39，51，58，68，66，	1	1	1、2、6	1	0	0	0	0
12110	38	1	-	1	1	7	0	0	0	0	0
12138	38	1	56,59,53	4	1	0	0	0	0	0	0
12162	38	1	33，52，68，42	1	2	0	2	0	0	0	0
11470	49	0		1	0	0	0			0	0
H-167	30	1	18、31	2	1	7	0	1	0	0	0
11472	43	1		1	1	5	0			0	0
12170	38	1	39,59	1	1	7	0	2	0	0	0
11474	26	0	52、58	1	0	6	1			0	0
H-008	38	1	18	1	2	2、5	4	0	0	0	0
11477	26	1		4	1	0	1			0	0
11478	47	0		1	0	6	6			0	0
11479	47	1	16	1	1	0	10			0	0
H-168	30	1	18	1	1	0	1	1	1	0	0

H-174	30	1	33、52	5	2	3	8	3	0	0	0
11483	45	0		1	0	1、6	0			0	0
11486	44	1		1	1	6	1			0	0
H-021	38	1	68	2	1	5	0	0	0	0	0
H-026	38	1	58、68	1	1	7	0	0	0	0	0
H-049	38	1	39	1	2	0	0	0	0	0	0
H-191	30	1	51	1	2	0	0	0	1	0	0
H-262	30	1	16、39、66	2	1	0	0	0	0	0	0
H-051	38	1	16	1	1	0	2	2	0	0	0
H-120	38	1	16	1	1	0	2	0	0	0	0
H-128	38	1	16	1	2	5	0	1	0	0	0
H-138	38	1	16	1	1	6	0	0	0	0	0
H-142	38	1	18、66	1	1	6	0	0	0	0	0
H-146	38	1	31、56	1	1	7	1	1	0	0	0
H-151	38	1	52	5	2	0	1	0	0	0	0
H-156	38	1	16	2	1	0	0	0	0	0	0

H-164	38	1	56	1	1	7	0	0	0	0	0
H-266	30	1	16	1	1	0	0	0	1	0	0
11802	44	0		1	0	0	1			0	0
H-271	30	1	52	1	1	2、3	5	2	1	0	0
H-201	38	1	16	4	1	0	0	0	0	0	0
H-229	38	1	51、42	2	1	0	0	0	0	0	0
H-288	30	1	51	4	1	0	0	0	0	0	0
H-294	30	1	18、52、39	4	1	2	1	0	0	0	0
H-254	38	1	33	1	2	7、1	1	0	0	0	0
H-259	38	1	52	1	1	3	8	3	0	0	0
H-260	38	1	16、51	4	1	0	0	1	0	0	1
H-298	30	1	59	4	1	1	4	4	1	0	0
12009	45	1	39，6	1	1	0	0	0	0	0	0
H-265	38	1	18	1	1	0	0	1	0	0	0
12011	24	1	52	3	1	2、7	2	0	0	0	0
12012	46	1	51.52.11	4	1	0	5	4	0	0	0

11009	39	0	-	1	0	1	1	0	0	0	0
11010	39	0	-	1	0	0	0	0	0	0	0
11018	39	0	56,58	1	0	0	0	0	0	0	0
11019	39	0	-	1	0	6	2	2	0	0	0
11025	39	0	66	1	0	7	0	0	0	0	0
12018	44	1	16，42	1	1	3	8	3	0	0	0
12019	49	1	59	2	1	1、2	1	0	0	0	0
11034	39	0	-	1	0	2、3	2	0	0	0	0
11098	31	0	58	1	0	6、7、3	3	2	0	0	0
12023	25	1	51.68.CP8304	4	1	4	0	0	0	0	0
12024	45	1	45	1	1	5	0	0	0	0	0
11107	31	0	-	1	0	0	4	2	0	0	0
12026	49	1	-	1	1	7	8	3	0	0	0
11214	31	1	31，68，42	1	1	6	0	0	0	0	0
11085	39	0	CP8304	1	0	2、5、6、8	1	0	0	0	0

11215	31	0	-	1	0	0	0	0	0	0	0
12031	24	1	39，68	4	1	0	2	2	0	0	0
11094	39	0	11，CP8304	1	0	1、2、6	1	0	0	0	0
11223	31	0		1	0	2、7	8			0	0
11232	31	0	66	1	0	6	0			0	0
12037	26	1	58，68，44	1	2	0	0	0	0	0	0
12038	46	1	56.42	1	1	0	0	0	0	0	0
12039	22	1	-	3	1	2	0	0	0	0	0
11096	39	0	51，11，CP8304	1	0	1、2、6	8	3	0	0	0
11233	31	0		1	0	0	0			0	0
11256	31	0	42	1	0	7	6			0	0
11311	31	0	35	1	0	6、3	8			0	0
11181	39	1	16	1	2	2、5	4	2	0	0	0
11311	31	0	35	1	0	6、3	8			0	0

11210	39	0	31	1	0	0	1	0	0	0	0
11314	31	1		2	1	2	1			0	0
11213	39	0	-	1	0	7	1	0	0	0	0
11216	39	1	-	1	1	1	1	0	0	0	0
12072	26	1	51,52,11，CP8304	1	1	2、5	0	0	0	0	0
12073	25	1	52	4	1	7	0	0	0	0	0
12074	25	1	51,11,53	1	1	0	2	1	0	0	0
11231	39	0	31，51，52	1	0	1、6	0			0	0
11314	31	1		2	1	2	1			0	0
11236	39	1	31	2	1	5	0			0	0
12078	43	1	52	1	1	0	1	0	0	0	0
11370	31	0		2	0	5	0			0	0
11801	31	0		1	0	0	4			0	1
11299	39	1		4	1	0	0			0	0
12082	43	1	31，53	1	1	0	1	0	0	0	0

11803	31	0		1	0	0	1			0	0
12084	24	1	68	1	1	2	2	0	0	0	0
12003	31	1	-	1	1	0	4	2	1	0	0
12086	24	1	51	5	2	6	0	0	0	0	0
12008	31	1	39	1	1	0	0	0	0	0	0
12022	31	1	51	1	2	8	1	0	0	0	0
11332	39	0		1	0	0	0			0	0
12076	31	1	52，58	3	2	1、4、7	1	0	0	0	0
12087	31	1	51,68,66,6	1	1	5、10	0	0	0	0	0
11357	39	1		1	2	0	1			0	0
11383	39	0		1	0	0	1			0	0
12094	45	1	51，58，68	2	1	6	1	0	1	0	0
12129	31	1	39	1	1	2	10	3	1	0	0
12144	31	1	52	1	1	0	0	0	0	0	0
12097	48	1	58	2	2	1、3、7	4	2	0	0	0
12155	31	1	-	1	1	2、9	1	0	0	0	0

12099	43	1	52,56,68	1	1	0	1	0	0	0	0
12183	31	1	-	1	1	0	0	0	0	0	0
12186	31	1	39	2	2	0	0	0	0	0	0
12102	48	1	51，68	1	1	1	0	0	0	0	0
11407	39	0		2	0	0	4			0	0
H-005	31	1	18	4	2	6	0	2	0	0	0
12105	24	1	33	4	2	0	0	0	0	0	0
11410	39	0		1	0	0	0			0	0
12107	21	1	59	1	1	2、3	3	0	0	0	0
H-054	31	1	16	1	1	0	1	0	0	0	0
H-063	31	1	45	2	1	0	0	0	0	0	0
11412	39	0	39	1	0	5	8			0	0
11419	39	0	52	1	0	5、6	8			0	0
12112	23	1	39，56，43	4	1	6	1	0	0	0	0
11466	39	0		1	0	0	1			0	0
12115	45	1	39	2	1	0	0	0	0	0	0

H-125	31	1	18、52	1	1	0	1	0	0	0	1
12118	21	1	16，51，43	1	1	9	0	0	0	0	0
H-182	31	1	58	2	1	2、7	0	0	0	0	0
11493	39	0		1	0	0	1			0	0
12121	45	1	31	1	1	3、5	5	4	0	0	0
12122	24	1	31	4	2	2	0	0	0	0	0
12002	39	1	16.51.58	1	1	7	1	0	1	0	0
H-190	31	1	16	1	1	7、4	1	0	1	0	0
12006	39	1	56	1	1	6	0	0	0	0	0
12126	43	1	45,52	1	2	0	1	0	0	0	0
12013	39	1	-	1	1	6	1	0	0	0	0
H-199	31	1	66	1	1	7	0	0	0	0	0
H-206	31	1	16	1	1	7	0	0	0	0	0
H-209	31	1	16、56	1	2	7	2	0	0	0	0
H-213	31	1	16	1	1	2	0	1	1	0	0
12065	39	1	51，53	1	1	6	3	0	0	0	0

H-218	31	1	52	2	2	7	1	0	0	0	0
12134	45	1	-	1	1	2	0	0	0	0	0
H-222	31	1	51、59、58	4	1	6	0	0	0	0	0
H-230	31	1	58	2	1	0	0	0	0	0	0
12071	39	1	56，58	1	1	0	0	0	0	0	0
12089	39	1	51，68，66	1	1	2、7、10	8	3	0	0	0
H-239	31	1	16	1	1	0	1	0	0	0	0
12140	26	1	35，39	4	1	5	1	0	0	0	0
12141	26	1	66	4	1	0	2	0	0	0	0
12127	39	1	51,52	1	1	10	1	0	0	0	0
12176	39	1	33	1	2	5	0	0	1	0	0
H-263	31	1	59	2	1	2	3	0	0	0	0
12145	45	1	39,51,58,11，CP8304	1	1	1、2	0	0	0	0	0
H-267	31	1	33、39	2	1	0	0	1	0	0	0

12177	39	1	56,44	2	1	5	0	0	0	0	0
12149	44	1	-	1	1	1、3	0	0	0	0	0
12150	24	1	-	1	1	5	0	0	1	0	0
H-270	31	1	59	2	1	2、3	6	2	0	0	0
12209	39	1	35	1	1	0	8	3	0	0	0
H-284	31	1	18、51	2	2	0	1	2	1	0	0
H-071	39	1	53	4	1	0	1	0	0	0	0
11033	32	0	-	1	0	0	0	0	0	0	0
H-080	39	1	16	1	1	0	0	0	0	0	0
H-084	39	1	16	1	1	0	1	0	0	0	0
12158	25	1	44	1	1	6	1	0	0	0	0
11037	32	0	-	1	0	0	0	0	0	0	0
H-094	39	1	59	1	1	0	0	0	0	0	0
H-099	39	1	12 other HR-HPV	1	1	2	2	0	0	0	0
H-112	39	1	58	1	1	6	5	4	0	0	0
11057	32	0	-	1	0	7	1	0	0	0	0

11078	32	0	11	1	0	0	0	0	0	0	0
12165	21	1	51	1	1	0	1	0	0	0	0
H-130	39	1	18	1	1	6	4	4	1	0	0
11106	32	0	-	1	0	6	0	0	0	0	0
11169	32	0	-	1	0	6、7、8、3	1	3	0	0	0
11173	32	1	51，6	4	1	7	0	0	0	0	0
H-160	39	1	16	1	1	6	0	0	0	0	0
H-175	39	1	16、45	5	2	0	4	2	1	0	0
12172	23	1	58	4	1	2	1	0	0	0	0
H-197	39	1	18	1	1	0	1	0	0	0	0
H-255	39	1	16	1	2	0	0	0	0	0	0
H-296	39	1	18	2	1	0	0	0	1	0	0
11011	40	0	-	1	0	0	0	0	0	0	0
11030	40	0	-	1	0	2、5	0	0	0	0	0
11212	32	0	-	1	0	0	1	0	0	0	0
11043	40	0	51,CP8304	1	0	1、7	1	0	0	0	0
11224	32	0		1	0	0	2			0	0

12181	43	1	58	4	1	0	0	0	0	0	0
11259	32	0		1	0	0	0			0	0
11297	32	0	56、44	1	0	7	1			0	0
12184	43	1	58	2	1	0	0	0	0	0	0
12185	43	1	66	1	1	6	0	0	0	0	0
11302	32	0		1	0	0	0			0	0
11327	32	0		1	0	2	1			0	0
11427	32	0		2	0	5	4			0	0
11062	40	0	-	1	0	0	2	2	0	0	0
12194	48	1	-	1	1	7	0	0	0	0	0
11479	32	0		1	0	0	8			0	0
11076	40	0	-	1	0	0	1	0	0	0	0
12200	24	1	-	1	1	5	0	0	0	0	0
11099	40	0	-	1	0	0	0	0	0	0	0
12027	32	1	39.52	1	1	4	1	0	0	0	0
11102	40	0	51,52,59,11<br>,CP8304	1	0	9、1、2、6	1	0	0	0	0

12211	46	1	39	1	1	7	0	0	0	0	0
12035	32	1	58.68	1	1	0	1	0	0	0	0
12061	32	1	52,58	1	1	6	1	0	0	0	0
12079	32	1	52	4	1	0	1	0	0	0	0
12101	32	1	51，68	1	1	0	0	0	0	0	0
12236	44	1	35	1	1	0	0	0	0	0	0
12104	32	1	58，6	4	2	1、10	1	0	0	0	0
12131	32	1	58	1	2	6	1	0	0	0	0
12163	32	1	68	2	1	0	0	0	0	0	0
12198	32	1	59	2	1	6	1	0	0	0	0
12213	32	1	66	2	1	0	0	0	0	0	0
BJYY00<br>6	50	1	51、66	4	1	7	2	0	1	0	0
11151	40	0	52，58，<br>68，6	1	0	6、10	1	2	0	0	0
BJYY00<br>9	45	1	56	4	1	7	1	0	0	0	0

11162	40	0	68，66	1	0	2	3	2	0	0	0
11230	40	0		1	0	0	0			0	0
11269	40	0		1	0	7	0			0	0
H-007	32	1	16	1	1	6	0	0	1	0	0
11309	40	0		1	0	0	1			0	0
11309	40	0		1	0	0	1			0	0
11347	40	0	39	1	0	6、3	5			0	0
11353	40	0	52	1	0	0	4			0	0
BJYY01											
8	48	1	16	1	2	7	1	3	0	0	0
BJYY01											
9	47	1	52	4	1	0	0	0	0	0	0
11369	40	0		1	0	6	5			0	0
H-010	32	1	58	1	1	6	2	2	0	0	0
11373	40	0		1	0	0	4			0	0
H-023	32	1	52	1	1	7	8	3	0	0	0
11401	40	0		1	0	6	4			0	0

11433	40	0		1	0	0	5			0	0
11438	40	1		1	1	1	0			0	0
H-036	32	1	39、52	1	1	5、6	1	0	0	0	0
BJYY02<br>8	50	0	-	1	0	2、7	1	0	0	0	0
11447	40	0		1	0	0	0			0	0
11800	40	0		1	0	0	0			0	0
H-052	32	1	58	1	2	7	5	3	0	0	0
BJYY03<br>3	46	0	58	1	0	7	1	0	1	0	0
12070	40	1	51,68,6	1	1	10	0	0	0	0	0
12111	40	1	58	4	2	6	0	2	0	0	0
H-074	32	1	16、18	1	1	7	0	0	0	0	0
12120	40	1	16,52,58	1	1	1、6	3	2	0	0	0
12125	40	1	-	2	1	5	1	0	0	0	0
H-089	32	1	51	2	2	2	0	0	0	0	0
12132	40	1	53	1	1	0	4	2	0	0	0

BJYY041	20	1	-	1	1	0	0	0	1	0	0
12142	40	1	-	4	1	5	1	1	0	0	0
H-107	32	1	51、58	2	1	5	0	0	0	0	0
H-126	32	1	16、52、58	1	2	0	0	2	0	0	0
12156	40	1	68,42	2	1	2	2	2	0	0	0
12179	40	1	58	1	1	1	1	2	0	0	0
H-171	32	1	16、68	1	2	0	0	2	0	0	0
H-003	40	1	58	1	1	7	0	2	0	0	0
BJYY049	19	1	-	1	1	0	5	4	0	0	0
BJYY050	44	1	16、42	1	1	3	8	3	1	0	0
H-083	40	1	16、12 other HR-HPV	1	2	1、7	6	2	1	0	0

BJYY052	43	1	16、11	1	1	7、1	8	3	0	0	0
BJYY053	45	1	11	1	1	6	3	2	0	0	0
H-105	40	1	52	1	1	6	0	0	0	0	0
H-133	40	1	16、58	1	1	10	0	0	0	0	0
BJYY056	50	1	16、11	1	1	6	1	0	0	0	1
H-134	40	1	52	2	1	0	0	0	0	0	0
BJYY058	47	1	53、11	1	1	0	0	0	0	0	0
BJYY059	24	1	52	1	1	2、7	2	0	0	0	0
BJYY060	46	1	51、52、11	4	1	0	5	4	0	0	0
H-157	40	1	68	1	1	6	1	0	0	0	0
H-172	32	1	16	1	1	0	0	2	0	0	0

H-203	40	1	56	4	1	1	0	0	0	0	0
BJYY064	43	1	52	1	1	7	0	1	1	0	0
BJYY065	43	1	51	1	1	0	1	0	1	0	0
H-185	32	1	52	4	1	1、7	2	2	0	0	0
H-300	40	1	59	1	1	7、9	1	0	1	0	0
H-217	32	1	33	4	1	7、1	8	3	0	0	0
BJYY069	26	1	35、39	4	1	5	5	3	0	0	1
BJYY070	44	1	51	1	1	5	4	3	0	0	0
BJYY071	45	1	16	1	1	0	0	0	0	0	0
11005	41	1	-	6	1	0	0	0	0	0	0
11014	41	1	-	1	1	0	1	0	1	0	0
11032	41	0	-	1	0	0	1	0	0	0	0

BJYY075	25	1	-	2	1	10、1	1	0	0	0	0
BJYY076	50	1	59	1	1	2、1	1	0	0	0	0
BJYY077	48	1	-	1	1	2、1	2	0	0	0	0
BJYY078	23	1	16、51、52、11	1	1	7	1	0	0	0	1
11051	41	1	-	1	1	0	0	0	0	0	0
H-249	32	1	52、58	2	1	1	1	0	0	0	0
11080	41	0	51，52，11	1	0	1、2、6	4	2	0	0	0
11089	41	0	58,11,CP8304	1	0	1、2	1	0	0	0	0
BJYY083	24	1	-	1	1	2、1	1	0	0	0	0

11127	41	1	39，45，51，68	1	1	6	0	2	1	0	0
11134	41	0	31	1	0	6	3	0	0	0	0
H-283	32	1	39、52、58	1	2	0	2	2	1	0	0
11006	33	0	-	1	0	2	4	4	1	0	0
11136	41	0	51	1	0	0	0	0	0	0	0
11013	33	0	-	1	0	0	0	1	0	0	0
BJYY090	22	1	16	1	1	1	8	3	0	0	0
11159	41	0	-	1	0	0	4	4	0	0	0
11193	41	0	43	1	0	7	3	2	1	0	0
11220	41	0	-	1	0	1	1	0	0	0	0
11345	41	0		1	0	0	0			0	0
BJYY095	49	1	11	1	1	1	1	0	0	0	0
11358	41	0		1	0	0	1			0	0

BJYY09<br>7	43	1	-	1	1	0	0	0	0	0	0
11366	41	0		1	0	0	0			0	0
11385	41	0		1	0	2	3			0	0
11022	33	0	-	1	0	0	1	0	0	0	0
11421	41	0		1	0	0	0			0	1
11054	33	0	52	1	0	0	0	0	0	0	0
11067	33	1	51，52，<br>11	1	1	1、2	4	2	0	0	0
11429	41	0		1	0	2、6	8			0	0
BJYY10<br>5	26	1	51、52、<br>11、<br>CP8304	1	1	2、5	0	0	0	0	0
11436	41	0		1	0	0	1			0	0
11112	33	0	-	1	0	0	0	0	0	0	0
11132	33	1	16，18，<br>51，68，6	1	1	10	1	0	0	0	0

11437	41	0		1	0	6	0			0	0
11444	41	0		1	0	2、6、7、3	6			0	0
BJYY11<br>1	47	1	-	1	1	0	0	0	0	0	0
11793	41	0	35、58	1	0	7	2			0	0
12021	41	1	58，66	2	1	0	1	0	0	0	0
12067	41	1	51	1	2	0	0	0	0	0	0
11139	33	0	68，6	1	0	10	0	0	0	0	0
BJYY11<br>6	24	0	39、68	1	0	0	2	2	0	0	0
12077	41	1	18	1	1	6	1	0	0	0	0
12103	41	1	-	1	1	0	0	0	0	0	0
BJYY11<br>9	25	0	-	1	0	2、3	8	3	0	0	0
BJYY12<br>0	43	1	33、39、<br>51、52、	2	1	7、1	4	2	1	0	0

			58、68、								
			11、								
			CP8304								
11189	33	0	58	1	0	1、6	0	1	0	0	0
11234	33	0		1	0	7	1			0	0
11258	33	0		1	0	0	4			0	0
11296	33	0	18	1	0	6	0			0	0
12137	41	1	6	1	1	7	0	0	0	0	0
BJYY12											
7	22	1	-	1	1	0	2	0	1	0	0
11307	33	0		1	0	0	3			0	0
BJYY12											
9	44	0	-	1	0	7	1	0	0	0	0
11307	33	0		1	0	0	3			0	0

BJYY13<br>1	43	0	39、52、<br>58、11、<br>CP8304	1	0	2	0	0	0	0	1
12154	41	1	16,33,68,42	4	1	7	2	2	0	0	0
12201	41	1	31	1	1	0	0	0	0	0	0
BJYY13<br>4	26	0	51、58、<br>11、<br>CP8304	1	0	5	0	0	0	0	0
BJYY13<br>5	49	1	18、31	1	1	7	0	0	0	0	0
BJYY13<br>6	47	0	-	1	0	0	1	0	0	0	0
11316	33	0		1	0	7	1			0	0

BJYY138	45	1	39、51、58、11、CP8304	2	1	2、1	0	0	0	0	0
H-053	41	1	12 other HR-HPV	2	2	2	8	3	1	0	0
BJYY140	24	0	39	1	0	2、5	3	0	0	0	0
H-095	41	1	33、51	1	1	6	0	0	0	0	0
11316	33	0		1	0	7	1			0	0
H-129	41	1	16、12 other HR-HPV	1	1	0	0	0	0	0	0
11349	33	0		1	0	5	4			0	0
H-135	41	1	56、66	4	2	0	3	0	0	0	0
H-145	41	1	12 other HR-HPV	1	1	1、5	0	1	1	0	0

BJYY14											
7	21	0	-	1	0	1	0	0	0	0	0
BJYY14											
8	43	1	-	1	1	0	0	0	0	0	0
11367	33	1		1	1	0	5			0	0
H-169	41	1	53	1	1	0	4	2	0	0	0
BJYY15											
1	47	1	16、42	1	1	6	0	0	0	0	0
H-275	41	1	52、61	1	1	7、3	0	0	0	0	0
BJYY15											
3	23	1	-	1	1	2、7	0	0	0	0	0
H-279	41	1	16、18	1	1	0	0	0	0	0	0
H-286	41	1	18	4	1	5	1	0	0	0	0
11020	42	0	-	1	0	0	0	0	0	0	0
11377	33	0	16	4	0	0	0			0	0
11791	33	0		1	0	2	0			0	0
11069	42	0	-	1	0	2	2	0	0	0	0

11084	42	0	-	1	0	8	0	0	0	0	0
12025	33	1	58	4	1	7	0	0	0	0	0
12083	33	1	56，58，CP8304	3	1	1	8	3	1	0	0
BJYY163	49	1	68、6	1	1	6、10	2	0	0	0	0
12088	33	1	51	2	1	0	0	0	0	0	0
BJYY165	48	1	45、51、52、58、68、6、11、CP8304	1	1	2、3、10	5	2	0	0	0
12100	33	1	68	2	1	0	0	0	0	0	0
12124	33	1	58	2	1	6	2	0	0	0	0
BJYY168	47	1	16、68、6	1	1	4、10	8	3	0	0	0

11161	42	1	39，58，6，44	1	1	7	0	0	0	0	0
12128	33	1	39	2	2	0	8	3	1	0	0
11178	42	1	39	1	1	2	0	0	0	0	0
BJYY172	25	1	-	1	1	0	2	0	0	0	0
12153	33	1	-	1	1	0	0	0	0	0	0
12159	33	1	52	4	1	7	4	3	1	0	0
BJYY176	45	1	-	1	1	0	0	0	0	0	0
BJYY177	49	1	51	1	1	5、3	8	3	1	0	0
11228	42	0		1	0	0	3			0	0
12168	33	1	51，39	1	1	2	0	0	0	0	0
11241	42	0		1	0	0	2			0	0
H-016	33	1	16、52	1	2	5	0	0	1	0	0
11249	42	1	16	1	1	2、3	3			0	0

11293	42	0		1	0	0	0			0	0
11318	42	0		1	0	0	0			0	0
H-043	33	1	16、18、45	5	2	1	0	1	0	0	0
11318	42	0		1	0	0	0			0	0
BJYY194	43	1	52	1	1	0	1	0	0	0	0
BJYY198	43	1	52、58、68、6、CP8304	3	1	7、10	8	3	0	0	0
11355	42	0		1	0	2	4			0	0
H-055	33	1	53	1	1	8	1	0	0	0	0
H-056	33	1	31、53	1	1	0	0	0	0	0	0
BJYY207	24	1	51	5	1	6	0	0	0	0	0
H-070	33	1	16、52	1	1	6	0	0	0	0	0
11413	42	1		1	1	3	8			0	0

11463	42	0		1	0	7	1			0	0
H-096	33	1	52	1	1	2	4	2	0	0	0
H-110	33	1	16	1	2	2、5、7	0	2	0	0	0
11491	42	0		1	0	6	1			0	0
BJYY224	49	1	CP8304	2	1	2、6	1	0	0	0	0
11798	42	0		1	0	6	0			0	0
12010	42	1	58,11	2	1	1、2、6	0	0	0	0	0
12029	42	1	58.CP8304	4	1	1、2	2	0	0	0	0
BJYY230	45	1	51、58、68	1	1	6	1	0	1	0	0
H-124	33	1	51、52、58	4	1	7、3	4	2	0	0	0
H-150	33	1	52	1	2	0	4	4	1	0	0
BJYY233	48	1	58	6	1	7、3、1	4	2	0	0	0
H-179	33	1	16、58	2	2	7	0	0	0	0	0

BJYY23			52、56、								
5	43	1	58	1	1	0	1	0	0	0	0
12092	42	1	33	4	1	1、6	0	0	0	0	0
BJYY23			35、39、								
7	23	1	51	1	1	0	5	2	0	0	1
12113	42	1	-	2	1	7	0	0	0	0	0
12148	42	1	-	3	1	0	1	0	0	0	0
12152	42	1	-	1	1	0	2	0	0	0	0
H-183	33	1	52	4	1	5	0	0	0	0	0
BJYY24											
2	44	1	6	1	1	7	3	3	0	0	0
H-189	33	1	18	5	2	7	0	0	1	0	0
H-193	33	1	18	1	1	0	0	0	0	0	0
BJYY24											
5	48	0	51、68	1	0	1	0	0	0	0	0
12160	42	1	33,58	1	1	7	0	0	0	0	0
H-207	33	1	16、59	1	1	6	5	2	0	0	0

BJYY248	24	1	33、45、58、68	4	1	0	0	0	0	0	0
12191	42	1	16	1	1	6、7	0	0	0	0	0
BJYY251	25	1	16、43、CP8304	1	1	4、1	4	2	0	0	0
H-062	42	1	12 other HR-HPV	2	1	5	0	0	0	0	0
BJYY254	21	1	-	1	1	2、3	3	0	0	0	0
H-219	33	1	58	1	1	0	1	0	0	0	0
11004	34	1	-	1	2	2	6	2	0	0	0
H-067	42	1	52	1	1	6	4	3	0	0	0
H-140	42	1	45	2	1	7	0	0	0	0	0
BJYY259	21	1	16、51、43	1	1	9	0	0	0	0	0
11028	34	0	-	1	0	5	0	0	0	0	0

BJYY261	44	1	52	2	1	2	3	2	0	0	0
H-149	42	1	12 other HR-HPV	4	2	7	0	0	0	0	0
H-195	42	1	56	1	1	7	0	0	0	0	0
BJYY264	45	1	31	1	1	5、3	5	4	0	0	0
BJYY265	24	1	31	1	2	2	0	0	0	0	0
H-205	42	1	52	1	2	0	7	4	0	0	0
11031	34	1	16	1	1	0	1	0	0	0	0
H-216	42	1	51	4	1	6	1	0	0	0	0
BJYY269	43	1	45、52	1	2	0	1	0	0	0	0
H-241	42	1	18	1	1	0	0	1	0	0	0
11059	34	0	11	1	0	0	4	2	1	0	0

BJYY27											
2	26	0	-	1	0	6、1	0	0	0	0	0
11060	34	0	-	1	0	0	1	0	1	0	0
11110	34	0	-	1	0	0	1	0	0	0	0
H-253	42	1	33	1	2	3	1	0	0	0	0
			35,39,51,58								
11117	34	0	,CP8304	1	0	7	1	0	0	0	0
11131	34	0	-	1	0	0	2	3	0	0	0
BJYY01											
5	35	1	-	1	1	3	5	5	1	0	0
BJYY02											
2	35	1	-	1	1	1	6	3	0	0	0
11138	34	0	68，6	1	0	9、10	0	0	0	0	0
BJYY28											
1	26	1	66	4	1	0	2	0	0	0	0
BJYY03											
8	35	1	-	4	1	0	0	0	1	0	0

BJYY28											
3	44	0	-	1	0	5	0	0	0	0	0
11217	34	0	-	1	0	6	0	0	0	0	0
BJYY04											
5	35	0	-	1	0	6	0	0	0	0	0
11219	34	0	-	1	0	0	3	2	1	0	0
11243	34	0		1	0	5	1			0	0
11270	34	0		1	0	0	1			0	0
11271	34	0		1	0	7	4			0	0
11273	34	0		1	0	0	3			0	0
11300	34	0		1	0	7	0			0	0
BJYY07											
3	35	1	16、18	1	1	9、7	0	0	0	0	0
11303	34	0		1	0	6	0			0	0
BJYY08											
4	35	1	42	1	1	2	1	0	0	0	0

BJYY10<br>1	35	1	-	1	1	0	0	0	0	0	0
BJYY12<br>6	35	1	16	1	1	6	1	0	1	0	0
BJYY13<br>2	35	1	16、39、<br>51、58、<br>6、11、<br>CP8304	1	2	2、1	0	0	0	0	0
BJYY14<br>6	35	1	52、58	1	1	7	1	0	0	0	0
11317	34	0	58、<br>CP8304	1	0	0	0			0	0
11317	34	0	58、<br>CP8304	1	0	0	0			0	0
BJYY21<br>4	35	0	-	1	0	0	3	2	0	0	0

BJYY30											
6	23	0	58	1	0	2	1	0	0	0	0
BJYY23											
8	35	1	58	1	1	0	0	0	0	0	0
11334	34	0		1	0	7	0			0	0
H-001	43	1	16	1	1	2、6	8	3	0	0	0
H-002	46	1	39	1	1	5	0	0	0	0	0
BJYY24											
9	35	0	56、68	1	0	7	6	2	0	0	0
11378	34	0		1	0	0	0			0	0
11388	34	0		1	0	0	1			0	0
H-006	45	1	高危型	4	2	5、3	2	0	0	0	0
11425	34	0		1	0	0	1			0	0
BJYY26											
6	35	1	-	1	1	0	1	0	0	0	0
11435	34	0		1	0	0	1			0	0
11471	34	0	53、42	1	0	5	0			0	0

H-011	25	1	16	1	2	7	0	0	0	0	0
BJYY29<br>7	35	0	58	1	0	6	0	0	0	0	0
BJYY30<br>0	35	1	56	1	1	2	0	0	0	0	0
H-014	25	1	18	1	1	6	8	4	0	0	0
BJYY04<br>6	36	1	16、39、<br>52、56	1	1	0	4	4	1	0	0
11480	34	0		1	0	6	0			0	0
H-017	44	1	16	1	1	2	0	0	0	0	0
BJYY08<br>1	36	1	-	1	1	0	8	3	0	0	0
12030	34	1	31	1	1	0	0	0	0	0	0
12063	34	1	52,68,6	1	1	10	0	0	0	0	0

BJYY118	36	1	39、68、11、CP8304	1	1	0	4	4	1	0	0
H-022	46	1	16、58	1	2	0	4	4	1	0	0
12064	34	1	16	1	1	0	1	0	0	0	0
12095	34	1	53	1	1	1	3	2	0	0	0
BJYY143	36	1	51、68、66	4	1	0	3	0	1	0	0
BJYY150	36	1	51、56、58、68、44、CP8304	1	1	0	1	1	0	0	0
12119	34	1	58	4	2	7	8	3	0	0	0
H-028	48	1	16	1	2	2、7	0	0	0	0	0
12205	34	1	59,68	1	1	7	1	0	0	0	0

H-009	34	1	12 other HR-HPV	1	1	6	0	0	0	0	0
H-031	48	1	53	1	1	3	5	2	0	0	0
H-019	34	1	66	1	1	7	3	0	0	0	0
H-030	34	1	52	1	1	0	4	2	1	0	0
BJYY159	36	1	45、51、52、58、68、6、11、CP8304	1	1	10	1	0	0	0	0
H-034	34	1	52	1	1	6	5	3	0	0	1
H-037	21	1	16、33	1	2	2	0	0	0	0	0
H-057	34	1	39、59、66	1	1	2、7	1	0	0	0	0
H-039	21	1	16、18	2	1	7	6	2	0	0	0
H-040	43	1	16、18	1	1	0	0	0	0	0	0

H-041	22	1	16、51、59	4	1	6	0	1	0	0	0
H-042	26	1	16、18	1	2	0	1	0	0	0	0
H-061	34	1	16、52、66	2	1	0	2	0	0	0	0
H-072	34	1	16、18	1	1	1、7	0	1	0	0	0
H-077	34	1	52	1	1	7	1	0	0	0	0
BJYY188	36	1	68、6	1	1	10	4	4	1	0	0
H-086	34	1	16	1	1	6	0	0	0	0	0
H-098	34	1	58	1	2	0	1	0	0	0	0
BJYY236	36	1	58、68	1	1	7、10、1	0	0	1	0	0
H-121	34	1	16	1	2	7	0	1	1	0	0
BJYY263	36	1	16、58、CP8304	1	1	0	1	0	1	0	0
H-122	34	1	53	2	1	7	0	1	0	0	0

BJYY30											
5	36	0	-	1	0	5	1	0	0	0	0
H-159	34	1	51	3	1	7、3	1	0	0	0	0
H-163	34	1	51	1	1	0	1	0	0	0	0
H-170	34	1	18、52	4	1	0	0	0	0	0	0
H-186	34	1	16	1	2	2、5	0	0	0	0	0
H-210	34	1	18	1	1	0	0	0	1	0	0
H-060	46	1	52、56	1	1	0	1	0	0	0	0
H-231	34	1	58	1	1	0	0	1	0	0	0
BJYY30											
7	36	0	68	1	0	6	2	0	0	0	0
H-278	34	1	53	4	1	0	8	3	0	0	0
H-064	44	1	16	1	1	6	7	2	1	0	0
BJYY08											
7	27	1	11	1	1	2、1	1	0	0	0	0
H-066	23	1	16、31	1	1	1	0	0	0	0	0

BJYY008	37	1	-	1	1	0	3	2	0	0	0
BJYY102	27	1	51、CP8304	1	1	0	0	0	0	0	0
H-069	43	1	31、56	1	1	6	0	2	0	0	0
BJYY204	27	0	31、39、51、66、6	1	0	2、10	1	1	0	0	1
BJYY010	37	1	-	1	1	0	2	0	1	0	0
BJYY256	27	0	31	1	0	0	0	0	0	0	0
BJYY280	27	1	16、58	1	1	1	2	0	1	0	0
BJYY100	28	1	51、11、CP8304	1	1	2、6、1	1	0	1	0	0

H-075	24	1	16、33、53	1	1	1	0	1	0	0	0
H-076	43	1	58	5	2	2	0	0	1	0	0
BJYY142	28	0	33、51、66	1	0	2、7、1	0	0	0	0	0
BJYY149	28	1	51	1	1	0	0	2	0	0	0
BJYY014	37	0	-	1	0	6、1	3	2	0	0	0
BJYY020	37	0	39	1	0	6	8	3	1	0	0
BJYY034	37	1	52	2	1	6	0	0	0	0	0
BJYY055	37	1	39、51、52、11	2	1	0	1	0	0	0	0

BJYY057	37	1	51	1	1	0	1	0	0	0	0
BJYY061	37	1	11	1	1	0	1	0	1	0	0
BJYY063	37	1	51	1	1	6	1	0	0	0	0
BJYY185	28	1	31、52、53、42、43	1	2	2、7	4	2	1	0	0
H-087	44	1	16	1	2	0	0	0	0	0	0
BJYY092	37	1	-	1	1	2、6、1	1	0	0	0	0
BJYY234	28	0	58	1	0	7	0	0	0	0	0
BJYY110	37	1	-	1	1	0	4	2	0	0	0
H-091	24	1	52	2	1	0	1	0	0	0	0

BJYY13<br>9	37	0	42	1	0	2、6、1	0	0	0	0	0
H-093	22	1	16、31、<br>45、39、<br>51	2	2	0	0	2	0	0	0
BJYY15<br>5	37	1	58、68、<br>11	1	1	0	3	2	0	0	0
BJYY18<br>9	37	0	6、66、<br>52、68	1	0	0	4	2	0	0	0
BJYY27<br>7	28	1	66	1	1	1	8	3	1	0	0
BJYY22<br>9	37	1	58	1	1	0	3	2	0	0	0
BJYY28<br>6	28	1	51	2	1	2、3	8	3	0	0	0

BJYY240	37	0	68	1	0	6、1	1	0	0	0	0
BJYY287	28	1	16	1	1	6	6	2	1	0	0
H-101	45	1	58	1	1	2	6	2	0	0	0
H-102	44	1	16	1	1	2	1	0	0	0	0
H-103	45	1	68	1	2	2、6	0	1	0	0	0
BJYY016	38	1	-	1	1	5	2	0	0	0	0
BJYY029	38	1	-	1	1	0	1	0	0	0	0
BJYY042	38	1	16、51、58	2	1	7	1	0	1	0	0
BJYY288	28	0	33、52、59	1	0	7	0	0	0	0	0
H-108	48	1	33	1	1	6	1	0	0	0	0

BJYY054	38	1	51、11	1	1	0	1	0	0	0	0
BJYY289	28	1	56、11	4	1	0	2	1	0	0	0
BJYY074	38	1	16、51、52、53、11	4	1	0	0	0	0	0	0
BJYY085	38	1	-	1	1	2、1	1	0	0	0	0
BJYY088	38	1	-	1	1	3	8	3	0	0	0
H-114	50	1	66	4	1	6、7	1	0	0	0	0
BJYY292	28	1	52	1	1	0	0	0	0	0	0
H-116	45	1	66、58	2	1	7、3	8	4	1	0	0
H-117	25	1	56	2	1	1、2	0	0	0	0	0

BJYY107	29	1	52、11、CP8304	1	1	2、1	5	3	1	0	0
BJYY183	29	1	39、52、68、6	1	1	5、10	1	0	0	0	0
BJYY093	38	1	-	1	1	0	1	0	0	0	0
BJYY218	29	1	51、52、58、6	1	1	0	0	0	0	0	0
BJYY004	30	1	-	1	1	2	0	0	0	0	0
H-123	44	1	16	1	2	2、6	0	0	0	0	0
BJYY023	30	0	-	1	0	7	0	0	1	0	0
BJYY044	30	1	56	3	1	3	4	2	0	0	0

BJYY08<br>6	30	1	51	1	1	4	0	1	0	0	0
H-127	47	1	56、58	4	1	7	2	0	0	0	0
BJYY09<br>8	38	1	51、11	1	1	7、1	1	0	0	0	0
BJYY11<br>3	38	1	-	1	1	7、1	4	4	0	0	0
BJYY11<br>7	38	0	39、51、<br>58、68、<br>66、11、<br>CP8304	1	0	2、6、1	1	0	0	0	0
BJYY14<br>1	38	0	33、51、<br>CP8304	1	0	2、6、1	0	0	0	0	0
BJYY10<br>3	30	1	-	1	1	5	0	0	0	0	0

BJYY156	38	1	39、45、51、58、68、6、11、42、CP8304	5	1	6、10	1	0	1	0	0
BJYY160	38	1	39	1	1	0	0	0	0	0	0
BJYY239	38	1	52	1	1	1	0	0	0	0	0
H-136	49	1	12 other HR-HPV	2	1	6	1	0	0	0	0
H-137	50	1	51	4	1	0	1	0	0	0	0
BJYY257	38	1	-	1	1	7	0	0	0	0	0
BJYY217	30	1	31、35、58	2	1	2、6、8、10	1	0	0	0	0

BJYY279	38	1	56、59、53	4	1	0	0	0	0	0	0
BJYY301	38	1	33、52、68、42	2	2	0	2	0	0	0	0
BJYY011	39	0	-	1	0	0	3	2	1	0	0
H-143	19	1	16	1	2	6、7	0	0	0	0	1
BJYY017	39	0	-	1	0	0	0	0	0	0	0
BJYY024	39	1	56、58	1	1	0	0	0	0	0	0
BJYY025	39	1	-	1	1	6	2	2	0	0	0
BJYY030	39	1	66	1	1	7	5	3	0	0	0
H-148	45	1	56	1	1	0	0	0	0	0	0

BJYY040	39	1	-	1	1	2、3	4	2	1	0	0
BJYY232	30	1	58、68	1	1	6、10、1	0	0	0	0	0
BJYY072	39	1	-	1	1	6	1	0	0	0	0
H-152	50	1	16、59	3	1	0	2	2	0	0	0
H-153	48	1	52	1	1	0	4	2	1	0	0
BJYY255	30	1	56、53	4	1	7	1	0	0	0	0
BJYY104	39	0	11、CP8304	1	0	2、6、1	1	0	0	0	0
BJYY106	39	0	51、11、CP8304	1	0	6、1	8	3	0	0	0
BJYY145	39	0	52	1	0	0	0	0	0	0	0

BJYY27<br>6	30	1	59	2	2	2	4	4	0	0	0
BJYY30<br>3	30	1	59	4	1	7	0	0	0	0	0
BJYY18<br>2	39	1	51、53	1	1	6	3	0	0	0	0
BJYY19<br>9	39	0	-	1	0	0	1	0	0	0	0
BJYY21<br>0	39	0	51、68、<br>66	1	0	2、7、10	8	3	0	0	0
BJYY30<br>9	30	0	52	1	0	6	0	0	0	0	0
BJYY22<br>5	39	1	16	1	2	2、5	4	2	0	0	0
BJYY04<br>3	31	1	-	1	1	0	4	2	1	0	0

BJYY047	31	1	39	2	1	0	0	0	0	0	0
BJYY108	31	0	58	1	0	6、7、3	3	3	0	0	0
BJYY125	31	1	-	1	1	0	4	2	0	0	0
BJYY270	39	1	51、52	1	1	10	1	0	0	0	0
BJYY170	31	1	-	1	1	0	0	0	0	0	0
BJYY190	31	0	52、58	1	0	7、4	1	0	0	0	0
BJYY208	31	1	51、68、66、6	1	1	5、10	0	0	0	0	0
H-173	24	1	16	1	2	0	0	0	0	0	0
BJYY273	31	1	39、68	1	1	6、3	0	0	0	0	0

BJYY285	39	0	-	1	0	5	0	0	0	0	0
H-176	25	1	12 other HR-HPV	4	2	6、3	8	3	1	0	0
H-177	45	1	16	2	1	0	1	0	0	0	0
BJYY299	39	1	31	1	2	0	1	0	0	0	0
BJYY284	31	1	16、53、68、51	1	1	0	0	0	0	0	0
BJYY035	40	1	-	1	1	2、5	0	0	0	0	0
BJYY051	40	1	51、CP8304	1	1	7、1	1	0	0	0	0
BJYY290	31	1	16、51、52、58	2	1	6、7	0	0	0	0	0

BJYY29<br>5	31	0	-	1	0	9、2	1	0	0	0	0
H-184	25	1	39	1	1	0	0	0	0	0	0
BJYY00<br>2	32	1	-	1	1	0	1	0	0	0	0
BJYY00<br>3	32	1	53	1	1	0	1	0	0	0	0
H-187	23	1	16	1	1	5	2	0	0	0	0
H-188	43	1	39、66	1	1	0	0	0	0	0	0
BJYY03<br>9	32	1	-	1	1	0	4	3	1	0	0
BJYY06<br>6	32	1	-	1	1	7	1	0	0	0	0
BJYY08<br>9	32	1	11	1	1	0	0	0	0	0	0
BJYY06<br>7	40	1	-	1	1	0	2	2	0	0	0

BJYY123	32	1	58、68	1	1	0	1	0	0	0	0
BJYY094	40	1	-	1	1	0	1	0	0	0	0
BJYY109	40	1	-	1	1	0	0	0	0	0	0
H-196	48	1	16	7	2	0	8	3	1	0	0
BJYY112	40	1	51、52、59、11、CP8304	1	1	9、2、6、1	1	0	0	0	0
BJYY152	40	1	52	1	1	0	0	0	0	0	0
BJYY124	32	1	-	1	1	6	0	0	0	0	0
H-200	46	1	16	1	1	0	0	0	0	0	0
BJYY186	40	1	51、68、6	1	1	10	0	0	0	0	0

H-202	48	1	12 other HR-HPV	2	1	0	1	0	0	0	0
BJYY262	40	1	16、52、58	1	1	6、1	3	2	0	0	0
H-204	21	1	18、51	1	1	7、4	5	3	1	0	0
BJYY268	40	0	-	1	0	5	1	0	0	0	0
BJYY157	32	1	68、6	1	1	6、10	0	0	0	0	0
BJYY161	32	1	52、58	2	1	6	1	0	0	0	0
H-208	49	1	18	1	2	0	1	0	0	0	0
BJYY162	32	1	31、59、6、CP8304	1	1	6	0	0	0	0	0
BJYY203	32	1	52	1	1	0	2	0	0	0	0

BJYY275	40	1	53	1	1	0	4	2	0	0	0
H-212	47	1	16	1	1	7	8	3	1	0	0
BJYY244	32	0	51、68	1	0	0	0	0	0	0	0
BJYY282	40	1	-	4	1	5	1	1	0	0	0
H-215	46	1	68	2	1	0	8	3	0	0	0
BJYY296	40	0	68、42	1	0	2	2	2	0	0	0
BJYY247	32	1	58、6	4	1	10、1	1	0	0	0	0
BJYY274	32	1	58	1	1	6	1	0	0	0	0
BJYY302	32	1	68	1	1	0	0	0	0	0	0

BJYY00<br>1	33	1	42、52	1	1	0	1	0	0	0	0
BJYY03<br>7	41	0	-	1	0	0	1	0	0	0	0
BJYY00<br>5	33	1	52	1	1	0	1	0	0	0	1
H-223	45	1	58	1	1	7、3	3	2	1	0	0
BJYY02<br>7	33	0	-	1	0	0	0	0	0	0	0
H-225	43	1	68、58	2	1	7、3	4	3	0	0	0
H-226	25	1	16、51、<br>39	1	1	5、6	1	0	0	0	0
H-227	23	1	16、33	1	1	3	8	3	0	0	0
H-228	45	1	18	1	1	6	0	0	0	0	1
BJYY07<br>9	41	1	58、66	1	1	0	1	0	0	0	0

BJYY03											
2	33	1	16	1	1	0	1	0	0	0	0
BJYY06											
2	33	1	52	1	1	0	0	0	0	0	0
H-232	44	1	16、53	1	1	6	0	0	0	0	0
H-233	25	1	39	1	1	0	1	1	0	0	0
H-234	24	1	16、35	1	2	0	1	0	0	0	0
BJYY08			51、52、								
0	33	1	11	1	1	2、1	4	2	0	0	0
BJYY13											
0	33	1	-	1	1	0	0	0	0	0	0
H-237	45	1	39	1	1	7、1	1	0	0	0	0
H-238	45	1	56	1	1	0	1	0	0	0	0
BJYY15											
8	33	1	6	1	1	0	4	2	0	0	0
H-240	44	1	16、81	4	2	0	0	0	0	0	0

BJYY091	41	1	51、52、11	1	1	2、6、1	4	2	0	0	0
H-242	22	1	39	2	1	2	0	0	0	0	0
H-243	47	1	56、58	4	1	7、3、2	4	2	1	0	1
H-244	20	1	12 other HR-HPV	2	1	7	0	0	0	0	0
BJYY099	41	1	58、11、CP8304	2	1	2、1	0	0	1	0	0
BJYY167	33	1	16、18、51、68、6、42、CP8304	1	1	10	1	0	0	0	0
BJYY133	41	1	39、51	1	1	5	0	0	0	0	0
BJYY174	33	1	68、6	1	1	10	0	0	0	0	0

BJYY24<br>1	33	1	58	1	1	6、1	0	1	0	0	0
H-250	45	1	18、39	1	1	0	0	0	1	0	0
BJYY24<br>3	33	1	68	2	1	0	0	0	0	0	0
BJYY15<br>4	41	1	39、45、<br>51、68	1	1	6	0	2	1	0	0
BJYY16<br>9	41	1	31	1	1	6	3	0	0	0	0
BJYY17<br>1	41	1	51	5	2	0	0	0	0	0	0
BJYY18<br>4	41	1	66、6	4	1	7	0	0	0	0	0
H-256	45	1	16	1	1	0	0	0	0	0	0
BJYY26<br>7	33	1	58	3	2	6	2	0	0	0	0
H-258	22	1	39	1	1	0	0	1	0	0	0

BJYY19											
1	41	1	18	1	1	6	1	0	0	0	0
BJYY24											
6	41	1	-	1	1	0	0	0	0	0	0
H-261	23	1	16、33	1	2	6、8	8	3	0	0	0
BJYY27											
1	33	1	39	2	1	0	8	3	1	0	0
BJYY01											
3	34	0	-	1	0	0	0	0	0	0	0
BJYY02											
1	34	0	68	1	0	0	1	2	0	0	0
BJYY25											
2	41	1	43	1	1	7	3	2	1	0	0
BJYY03											
6	34	1	16	1	1	0	0	0	0	0	0
BJYY06											
8	34	1	11	1	1	0	4	2	1	0	0

BJYY258	41	1	16、52、58、6、43	3	1	1	0	0	0	0	0
H-269	24	1	16、51	1	1	1	10	3	1	0	0
BJYY115	34	1	31	1	1	0	0	0	0	0	0
BJYY122	34	1	33、39、52、68、11、CP8304	1	1	2、5、7	2	0	0	0	0
H-272	25	1	16、58	4	2	7	0	0	0	0	0
H-273	46	1	16、52	1	2	7、3	6	2	1	0	0
H-274	47	1	58	1	1	2	3	0	1	0	0
BJYY278	41	1	6	1	1	7	0	0	0	0	0
BJYY128	34	1	-	1	1	0	1	0	0	0	0

H-277	49	1	53	2	1	6	2	0	0	0	0
BJYY13<br>7	34	0	35、39、<br>51、58、<br>CP8304	1	0	7	1	0	0	0	0
BJYY29<br>4	41	1	16、33、<br>68、42	1	1	7	2	2	0	0	0
BJYY01<br>2	42	1	-	1	1	0	0	0	0	0	0
H-281	50	1	66	1	1	5	1	0	0	0	0
H-282	25	1	12 other<br>HR-HPV	1	1	5	0	0	0	0	0
BJYY14<br>4	34	0	51、68、<br>53、11、<br>CP8304	1	0	0	0	0	0	0	0

BJYY16<br>4	34	1	52、68、6	1	2	10	0	0	0	0	0
H-285	26	1	16、18	4	2	1、3	8	3	0	0	0
BJYY02<br>6	42	0	-	1	0	6	0	0	0	0	0
H-287	49	1	52	2	1	0	3	0	0	0	0
BJYY16<br>6	34	1	-	1	1	0	2	3	0	0	0
H-289	25	1	16	1	1	3、2	7	4	1	0	0
BJYY04<br>8	42	1	16、56、<br>58、11	2	1	2、6、1	0	0	0	0	0
BJYY08<br>2	42	1	-	1	1	2	2	0	0	0	0
H-292	47	1	16	2	1	7	2	0	1	0	0
BJYY09<br>6	42	1	-	1	1	8	0	0	0	0	0

BJYY17<br>3	34	1	68、6	1	1	10	0	0	0	0	0
BJYY11<br>4	42	1	58、<br>CP8304	4	1	2、1	2	0	0	0	0
BJYY22<br>2	42	1	39	1	1	2	0	0	0	0	0
BJYY23<br>1	34	1	35、58、<br>68、53、6	1	1	1	3	2	0	0	0
BJYY26<br>0	34	1	58	4	2	0	8	3	1	0	0
H-299	24	1	66、68、<br>58	1	1	0	0	1	0	0	0
BJYY22<br>8	42	1	33	4	1	6、1	0	0	0	0	0
